# Nicotinamide Adenine Dinucleotide Phosphate Oxidase–Mediated Redox Signaling and Vascular Remodeling by 16α-Hydroxyestrone in Human Pulmonary Artery Cells: Implications in Pulmonary Arterial Hypertension

**DOI:** 10.1161/HYPERTENSIONAHA.116.07668

**Published:** 2016-08-10

**Authors:** Katie Y. Hood, Augusto C. Montezano, Adam P. Harvey, Margaret Nilsen, Margaret R. MacLean, Rhian M. Touyz

**Affiliations:** From the Institute of Cardiovascular and Medical Sciences, University of Glasgow, United Kingdom.

**Keywords:** estrogens, hypertension, pulmonary, models, animal, NADPH oxidase, superoxide

## Abstract

Estrogen and oxidative stress have been implicated in pulmonary arterial hypertension (PAH). Mechanisms linking these systems are elusive. We hypothesized that estrogen metabolite, 16α-hydroxyestrone (16αOHE1), stimulates nicotinamide adenine dinucleotide phosphate oxidase (Nox)–induced reactive oxygen species (ROS) generation and proliferative responses in human pulmonary artery smooth muscle cells (hPASMCs) and that in PAH aberrant growth signaling promotes vascular remodeling. The pathophysiological significance of estrogen–Nox–dependent processes was studied in female Nox1^−/−^ and Nox4^−/−^ mice with PAH. PASMCs from control subjects (control hPASMCs) and PAH patients (PAH-hPASMCs) were exposed to estrogen and 16αOHE1 in the presence/absence of inhibitors of Nox, cytochrome P450 1B1, and estrogen receptors. Estrogen, through estrogen receptor-α, increased Nox-derived ROS and redox-sensitive growth in hPASMCs, with greater effects in PAH-hPASMCs versus control hPASMCs. Estrogen effects were inhibited by cytochrome P450 1B1 blockade. 16αOHE1 stimulated transient ROS production in hPASMCs, with sustained responses in PAH-hPASMCs. Basal expression of Nox1/Nox4 was potentiated in PAH-hPASMCs. In hPASMCs, 16αOHE1 increased Nox1 expression, stimulated irreversible oxidation of protein tyrosine phosphatases, decreased nuclear factor erythroid–related factor 2 activity and expression of nuclear factor erythroid–related factor 2–regulated antioxidant genes, and promoted proliferation. This was further amplified in PAH-hPASMCs. Nox1^−/−^ but not Nox4^−/−^ mice were protected against PAH and vascular remodeling. Our findings demonstrate that in PAH-hPASMCs, 16αOHE1 stimulates redox-sensitive cell growth primarily through Nox1. Supporting this, in vivo studies exhibited protection against pulmonary hypertension and remodeling in Nox1^−/−^ mice. This study provides new insights through Nox1/ROS and nuclear factor erythroid–related factor 2 whereby 16αOHE1 influences hPASMC function, which when upregulated may contribute to vascular injury in PAH, particularly important in women.

## Abstract 2

Supplemental Digital Content is available in the text.

The prevalence of pulmonary arterial hypertension (PAH) is greater in women than in men. Exact reasons for this sex-related difference remain unclear although increasing evidence suggests that metabolites of 17β-estradiol (estrogen) may play a role.^1–3^ Despite the female predominance and pathological implications of estrogens in human PAH, some experimental models have shown that exogenous estrogen is protective.^4^ The apparent contradictions may be explained by differential effects of estrogen metabolites on pulmonary vascular function and right ventricular (RV) homeostasis.^5,6^

Estrogen is metabolized by cytochrome P450 (CYP) enzymes^7,8^ to both proproliferative and antiproliferative metabolites.^9^ CYP 1B1 (CYP1B1) is a P450 enzyme expressed in the lung, which catalyses the conversion of estrogens predominantly to 4-hydroxyestrogens but also to 2-hydroxy and 16-hydroxyestrogens,^10^ and has been implicated in idiopathic PAH^1^ and heritable PAH.^3,11,12^ 16α-hydroxyestrone (16αOHE1) is a biologically active metabolite with estrogenic activity. It is more potent than that of estrogen.^13^ 16αOHE1 stimulates cell proliferation and has been implicated in experimental PAH,^1^ as well as having genotoxic effects in other systems.^14^ Molecular processes underlying these effects are unclear, although reactive oxygen species (ROS) may be important because estrogen, through its estrogen receptor (ER), and estrogen metabolites have been shown to cause cell proliferation through redox-sensitive processes.^15^

Increased bioavailability of ROS (superoxide anion [^·^O_2_^**−**^]) hydroxyl radical and hydrogen peroxide [H_2_O_2_]) leads to a shift in the balance between pro-oxidants and antioxidants and has been implicated in the development of various cardiovascular diseases, including PAH.^16–18^ The nicotinamide adenine dinucleotide phosphate oxidase (Nox) family of enzymes is the primary source of ROS production in the vasculature, where increased expression of Nox isoforms 1 and 4 in the pulmonary vasculature has been demonstrated in experimental models of PAH.^16–18^

In support of the importance of Noxs in PAH, studies in rat models demonstrated that antioxidants, such as resveratrol analogs, improved pulmonary hypertension^16^ and that in mice, mitochondria-localized Nox4 activity is increased in the early phase of pulmonary hypertension.^17^ Moreover, in pulmonary artery smooth muscle cells (PASMCs) isolated from monocrotaline-induced pulmonary hypertension, activation of Nox1, but not Nox4, was increased and Nox1-dependent signaling pathways were upregulated.^18^ Counter-regulating pro-oxidants in vascular cells are antioxidants; many of which are controlled by nuclear factor erythroid–related factor 2 (Nrf2), a key transcription factor that influences activation of antioxidant genes, such as superoxide dismutase (SOD), catalase, and thioredoxin, which protect against oxidative damage.^19^ Although oxidative stress may be important in the pathophysiology of PAH, the relationship to estrogen and its metabolites and the potential significance in women remain unclear.

We hypothesized that 16αOHE1 stimulates Nox-induced ROS generation and proliferative responses in PASMCs and that in PAH, Nox dysregulation, and Nrf2 downregulation leads to aberrant mitogenic signaling and increased cell proliferation, which are important in vascular remodeling in PAH. Because of the preponderance of PAH in women, we focused our study on PASMCs from well-characterized female patients. To investigate the pathophysiological significance of estrogen–Nox–dependent processes in PAH, we studied female Nox1^−/−^ and Nox4^−/−^ mice with pulmonary hypertension.

## Materials and Methods

A detailed Methods section is provided in the online-only Data Supplement.

### Cell Culture

In vitro studies were performed using primary cultures of human PASMCs (hPASMCs) from small distal arteries of the pulmonary vasculature from well-characterized female PAH patients (PAH-hPASMCs) and control subjects without PAH (control hPASMCs; provided by N. Morrell, University of Cambridge, Cambridge, United Kingdom). Patient details are shown in Table S1 in the online-only Data Supplement. As comparator cells, in some experiments, we also studied human vascular SMCs (hVSMCs) from peripheral arteries obtained from gluteal biopsies of healthy women. Cells were used between passages 3 and 6 and processed as we described.^20^ Experimental procedures using hPASMCs conform to the principles outlined in the Declaration of Helsinki and were approved by Cambridgeshire 1 Research Ethics Committee (REC reference: 08/H0304/56).

### Cell Protocols

Cells were stimulated with estrogen or 16αOHE1 (1 nmol/L; 5 minutes to 48 hours). In some protocols, cells were pretreated (30 minutes) with pharmacological inhibitors: 2-acetylphenothiazine (ML171; Nox1 inhibitor, 1 µmol/L), GKT137831 (Nox1/4 inhibitor, 1 µmol/L), gp91ds-tat (Nox2 inhibitory peptide or scrambled control peptide, 10 µmol/L), 1,3-bis(4-hydroxyphenyl)-4-methyl-5-[4-(2-piperidinylethoxy) phenol]-1*H*-pyrazole dihydrochloride (MPP; ERα antagonist, 100 nmol/L), 4-[2-phenyl-5,7-bis(trifluoromethyl)pyrazolo[1,5-*a*]pyrimidin-3-yl]phenol (PHTPP; ERβ antagonist, 100 nmol/L), 4-hydroxy-2,2,6,6-tetramethylpiperidine 1-oxyl (tempol; SOD mimetic, 10 µmol/L), and 2,3′,4,5′-tetramethoxystilbene (TMS; CYP1B1 inhibitor, 100 nmol/L). Doses were based on preliminary experiments and published data as detailed in the online-only Data Supplement.

### Lucigenin-Enhanced Chemiluminescence

Lucigenin-enhanced chemiluminescence was used to determine ROS generation in cell lysates as we described.^20,21^

### Amplex Red Assay

H_2_O_2_ was assessed in cell lysates with Amplex Red assay kit according to manufacturer’s instructions.

### Immunoblotting

Immunoblotting was used to examine protein expression of proliferating cell nuclear antigen, p27, Nox1 and Nox4, CYP1B1 and activation of signaling protein, p38mitogen-activated protein kinase, and irreversible oxidation of protein tyrosine phosphatases (PTPs).

### PTP Oxidation

Irreversible oxidation of PTPs was assessed using an antibody (anti-Ox-PTP) that specifically recognizes the sulfonic acid form of PTP cysteine residues as described.^22^

### Real-Time Polymerase Chain Reaction

Quantitative real-time polymerase chain reaction was used to analyze mRNA expression. Total RNA was extracted, and real-time quantitative polymerase chain reaction was carried out using SYBR Green I as described.^23^ Primers used were designed using the software Primer 3 online (Table S2).

### Nrf2 Activity Assay

Nrf2 activity was determined with the TransAM Nrf2 assay following manufacturer’s instructions.

### 5-Bromo-2′-Deoxyuridine Incorporation Assay

Cell proliferation was measured by 5-bromo-2′-deoxyuridine incorporation.

### Hypoxic-Induced Pulmonary Hypertension in Female Nox1^−/−^ and Nox4^−/−^ Mice

All animal procedures conform to the UK Animal Procedures Act (1986), ARRIVE Guidelines,^24^ and the Guide for the Care and Use of Laboratory Animals (National Institutes of Health publication number, 85-23, revised 1996). Nox1^−/−^ and Nox4^−/−^ mice have previously been described.^25,26^ Mice were given free access to regular chow diet and water and were maintained on a 12-hour light/dark cycle. Development of hypoxic pulmonary hypertension in 18-week-old female Nox1^−/−^ and Nox4^−/−^ mice and age-matched wild-type (WT) female littermates (strain C57BL/6J) was achieved by 15 days exposure to hypobaric hypoxia (10% O_2_; 550 mbar) as described.^27^ Mice maintained in normoxic conditions (21% O_2_; 1013 mbar) were studied as controls.

### In Vivo Assessment of Pulmonary Hypertension

For all in vivo procedures, mice were assessed at 20 weeks of age and were anesthetized with inhaled isoflurane (3% in O_2_, induction; 1.5% in O_2_, maintenance). In vivo pressure–volume loop relation measurements were performed to assess hemodynamic alterations in anesthetized mice 15 days after exposure to hypoxic conditions. A pressure–conductance catheter was inserted in the RV via the right jugular vein for right-heart catheterization and via the carotid artery for left ventricular catheterization (Millar Instruments, Houston, TX). After stabilization, steady-state measurements were recorded. RV systolic pressure (RVSP), RV end-systolic pressure, RV end-diastolic pressure, left ventricular systolic pressure, mean arterial pressure, heart rate, stroke volume, and cardiac output were determined.^28–30^ RV hypertrophy (RVH) was assessed by bisecting the heart into the RV and left ventricle (LV) plus septum. RV and LV+septum ratio were determined (RV/[LV+septum]). Left ventricular hypertrophy was determined as left ventricular weight:tibia length.

### Lung Immunohistochemistry to Assess Pulmonary Vascular Remodeling

Immediately after harvest, the left lung was perfusion fixed via the trachea. Lungs were processed into paraffin blocks for sectioning and stained for elastin and collagen using Van Gieson and Picrosirius red, respectively.^27^

### Statistical Analysis

Mean value±SEM were calculated, and statistical comparisons were made with 1-way or 2-way ANOVA followed by Tukey post hoc test or 2-tailed Student *t* test where appropriate. *P*<0.05 was considered statistically significant.

## Results

### Estrogen and 16αOHE1 Increase ROS Production Through Nox

**Figure 1.**
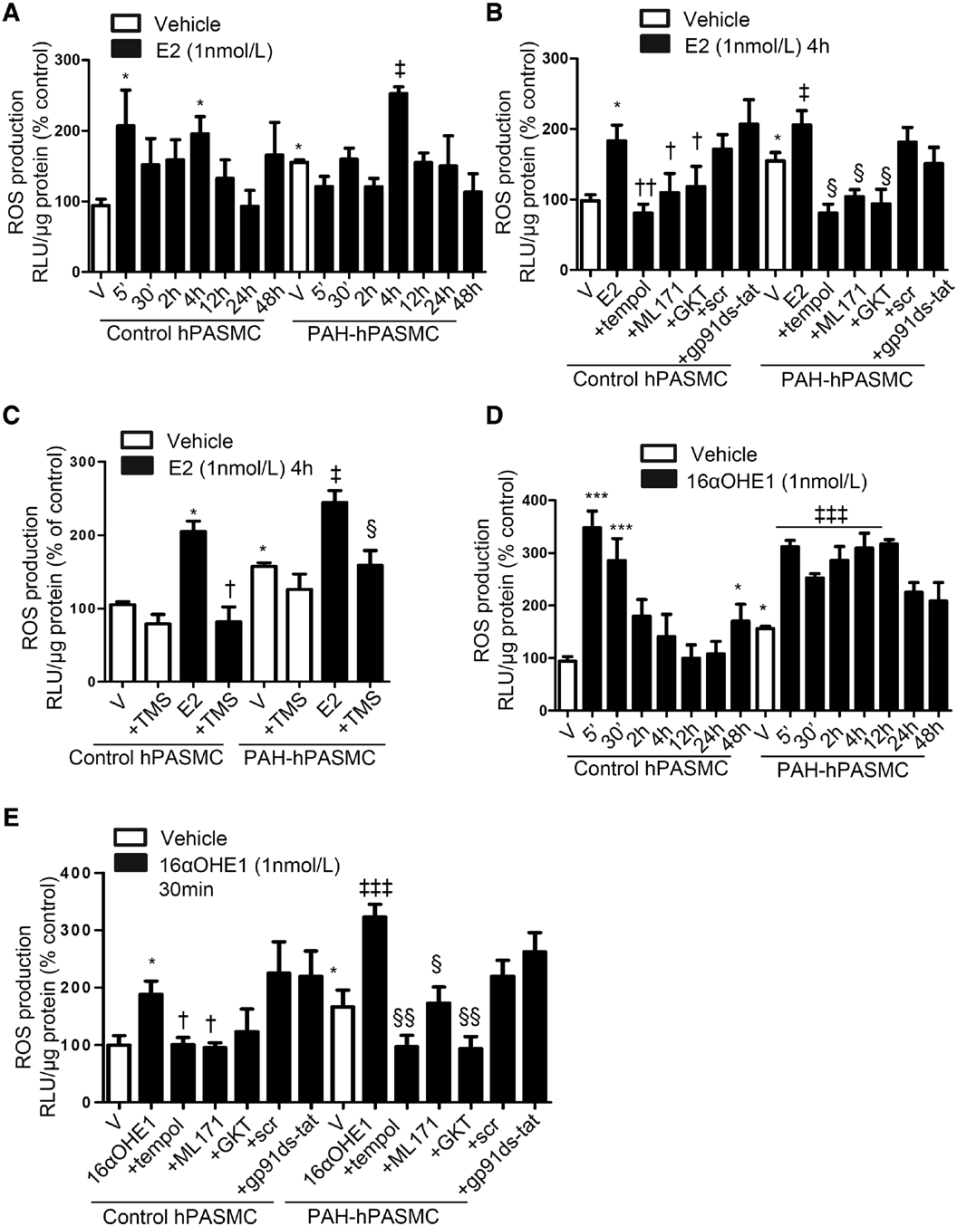
Estrogen (E2) and 16α-hydroxyestrone (16αOHE1) increase reactive oxygen species (ROS) production through nicotinamide adenine dinucleotide phosphate oxidase (Nox)-dependent mechanisms. Time-dependent increase of ROS production by E2 (1 nmol/L) in control human pulmonary artery smooth muscle cells (hPASMCs) and pulmonary arterial hypertension (PAH)-hPASMCs (**A**). **A**, hPASMCs and PAH-hPASMCs were exposed to E2 for the time of peak ROS production (4 h), in the presence or absence of inhibitors of Nox1 (ML171, 1 µmol/L), Nox1/4 (GKT137831, 1 µmol/L), and Nox2 (gp91ds-tat or peptide control scrambled gp91ds-tat control peptide [Scr], 10 µmol/L). **B**, Cells were also exposed to the superoxide dismutase mimetic tempol (10 µmol/L). **C**, E2-induced ROS production in the presence of cytochrome P450 1B1 inhibitor, 2,3′,4,5′-tetramethoxystilbene (TMS; 100 nmol/L). **D**, Time-dependent increase of ROS production by 16αOHE1. **E**, 16αOHE1-induced ROS production at peak time point, 30 minutes, in the presence or absence of tempol, ML171, GKT137831, or gp91ds-tat in control hPASMCs and PAH-hPASMCs. Data are expressed as relative light units (RLUs)/µg protein, expressed as percentage of vehicle (V) control conditions. Results are presented as mean±SEM of 6 to 7 experiments in triplicate. *P<0.05 and ***P<0.001 vs vehicle control hPASMCs; †*P*<0.05 and ††*P*<0.01 vs treated control hPASMCs; ‡*P*<0.05 and ‡‡‡*P*<0.001 vs vehicle PAH-hPASMCs; §*P*<0.05 and §§*P*<0.01 vs treated PAH-hPASMCs determined by ANOVA with Tukey post hoc test.

Basal ROS production was higher in PAH-hPASMCs compared with control hPASMCs (Figure 1A). In control hPASMCs, estrogen induced a biphasic ROS response, with a rapid increase at 5 minutes and a second peak at 4 hours. In PAH-hPASMCs, estrogen induced a significant increase in ROS generation at 4 hours (Figure 1A). Estrogen-induced ROS production was blocked by ML171, a Nox1 inhibitor, and GKT137831, a dual Nox1/Nox4 inhibitor and the ROS scavenger, tempol (Figure 1B). The specific peptide inhibitor of Nox2, gp91ds-tat, did not inhibit ROS production (Figure 1B).

Estrogen can be converted to 16αOHE1 by CYP1B1. 2,3′,4,5′-tetramethoxystilbene, a selective CYP1B1 inhibitor, blocked estrogen- but not 16αOHE1-induced ROS production in control hPASMCs and PAH-hPASMCs (Figure 1C; Figure S1). 16αOHE1 induced a rapid, but transient, increase in ROS generation in control hPASMCs, whereas in PAH-hPASMCs, effects were sustained (Figure 1D). 16αOHE1-stimulated ROS formation was inhibited by tempol (SOD mimetic), ML171, and GKT137831 (Figure 1E). No effects on ROS production were observed with the inhibitors alone (data not shown).

**Figure 2.**
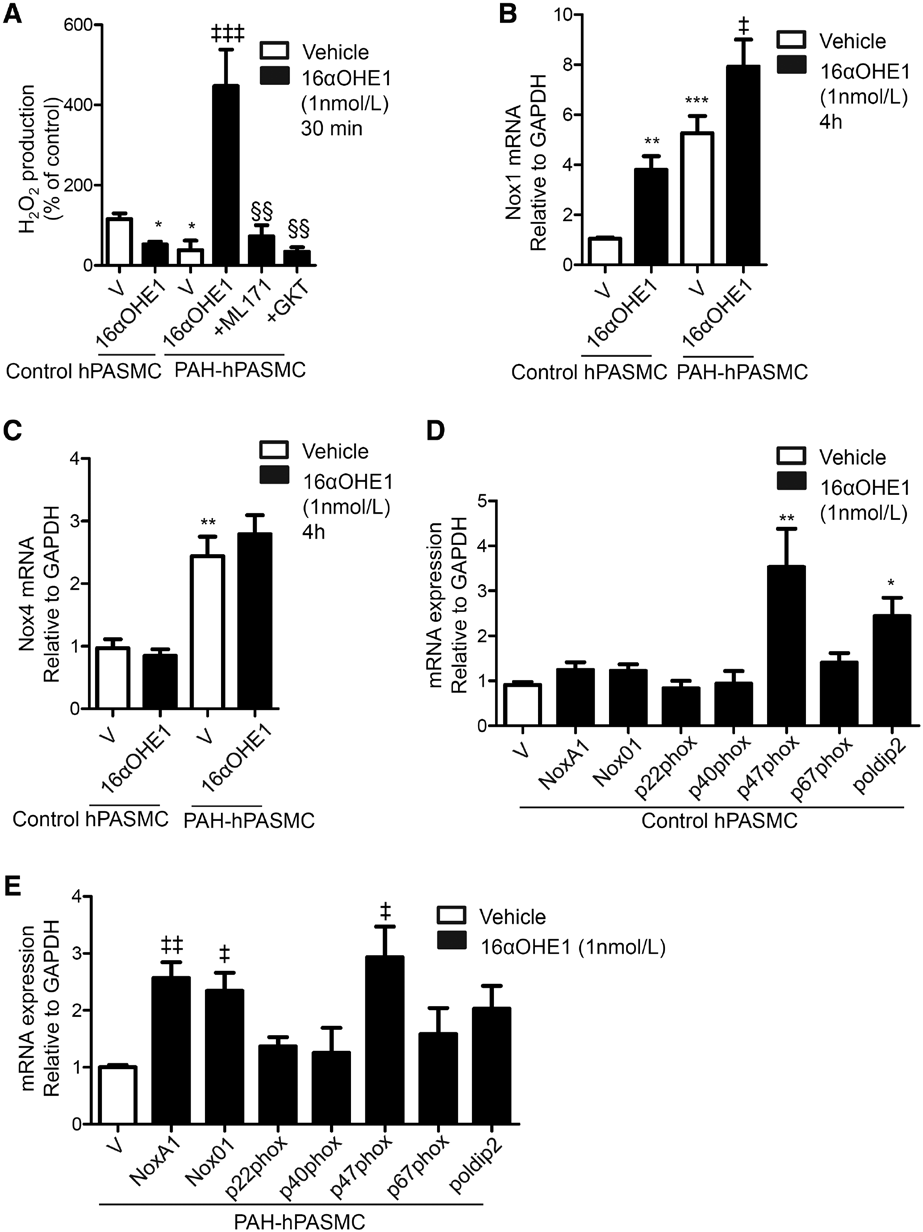
Effect of 16α-hydroxyestrone (16αOHE1) on hydrogen peroxide (H_2_O_2_) production and nicotinamide adenine dinucleotide phosphate (NADPH) oxidase isoform expression. **A**, H_2_O_2_ production from cell lysates was measured by using Amplex Red assay in cells exposed to 16αOHE1 for 30 minutes in the presence or absence of ML171 and GKT137831. Data are expressed as relative light units (RLUs)/µg protein corrected to standard curve and expressed as percentage of vehicle (V) control conditions. Transcript levels of nicotinamide adenine dinucleotide phosphate oxidase (Nox)-1 (**B**) and Nox4 (**C**) and NADPH oxidase regulatory proteins in response to 16αOHE1 (4 h) in control human pulmonary artery smooth muscle cells (hPASMCs) (**D**) and pulmonary arterial hypertension (PAH)-hPASMCs (**E**). Results are presented as mean±SEM of 6 experiments in triplicate. Graphs represent mRNA expression relative to GAPDH. *P<0.05, **P<0.01, and ***P<0.001 vs vehicle control hPASMCs; ‡*P*<0.05, ‡‡*P*<0.01, and ‡‡‡*P*<0.001 vs vehicle PAH-hPASMCs; §§*P*<0.01 vs 16αOHE1-treated PAH-hPASMCs, determined by ANOVA with Tukey post hoc test.

Basal H_2_O_2_ levels were reduced in PAH-hPASMCs versus control hPASMCs. 16αOHE1 decreased H_2_O_2_ production in control hPASMCs but markedly increased H_2_O_2_ levels in PAH-hPASMCs (Figure 2A). This may be via Nox1 and Nox4 as H_2_O_2_ production was inhibited by the Nox inhibitors, ML171 and GKT137831 (Figure 2A).

We next investigated the ER receptor subtype, mediating 16αOHE1 ROS effects. The ERα antagonist, 1,3-bis(4-hydroxyphenyl)-4-methyl-5-[4-(2-piperidinylethoxy) phenol]-1*H*-pyrazole dihydrochloride, but not the ERβ antagonist, 4-[2-phenyl-5,7-bis(trifluoromethyl)pyrazolo[1,5-*a*]pyrimidin-3-yl]phenol, inhibited 16αOHE1-induced ROS formation in control and PAH-hPASMCs (Figure S2). No effects were observed with the ER antagonists alone (data not shown).

### Regulation of Nox Isoforms and Nox Subunits by 16αOHE1

Basal gene expression of Nox1 and Nox4 was increased in PAH-hPASMCs compared with control hPASMCs (Figure 2B and 2C). Nox2 transcript expression in both control hPASMCs and PAH-hPASMCs was below reliable levels of detection (data not shown). 16αOHE1 increased Nox1 expression in control hPASMCs to levels observed in PAH-hPASMCs (Figure 2B). In control hPASMCs, 16αOHE1 also increased gene expression of p47phox, the Nox subunit capable of activating Nox2 and Nox1 in the hybrid system in lieu of NoxO1, and poldip2, a Nox4 regulatory protein (Figure 2D). In PAH-hPASMCs, 16αOHE1 increased transcript levels of NoxA1, NoxO1, and p47phox, subunits that regulate Nox1 (Figure 2E). Protein expression of Nox1 and Nox4 was increased in PAH-hPASMCs compared with control hPASMCs, which is in agreement with transcript expression. Nox1, but not Nox4, protein levels were further increased after 16αOHE1 treatment in PAH-hPASMCs (Figure S3).

### Regulation of Nrf2 and Antioxidant Systems by 16αOHE1

**Figure 3.**
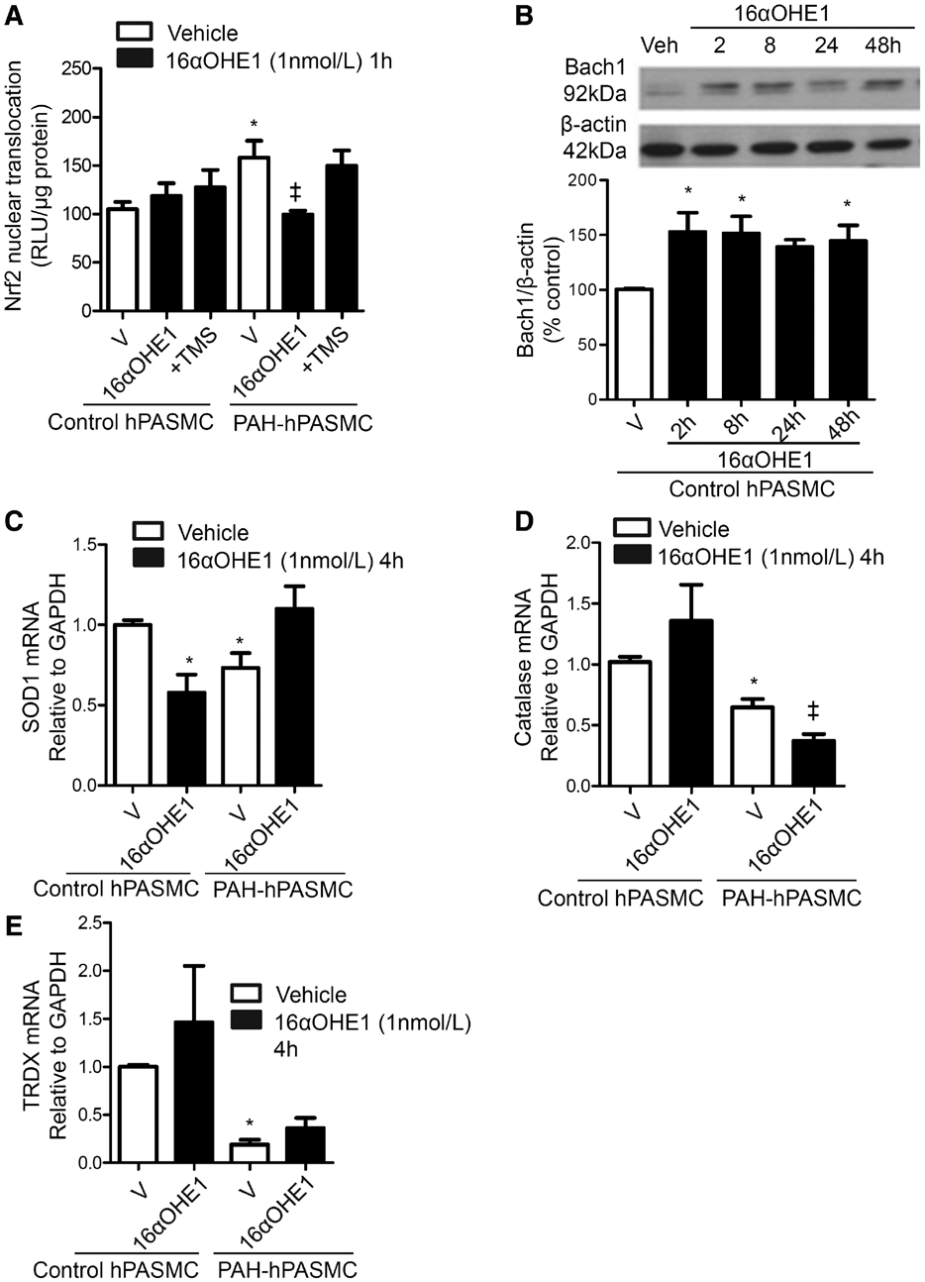
Effect of 16α-hydroxyestrone (16αOHE1) on nuclear factor E2–related factor 2 (Nrf2) activation and antioxidant gene expression. Nuclear translocation of Nrf2 by 16αOHE1 at 1 h was assessed as an indicator of Nrf2 activity, in the presence of cytochrome P450 1B1 inhibitor, 2,3′,4,5′-tetramethoxystilbene (TMS). Data are expressed as relative light units (RLUs)/µg protein expressed as percentage of vehicle control conditions. **A**, Results are presented as mean±SEM of 6 experiments in triplicate. Effects of 16αOHE1 (2–48 h) on protein expression of Nrf2 transcriptional repressor, Bach1 (BTB and CNC homology 1). **B**, Protein expression is relative to β-actin. Antioxidant responses were assessed by investigating antioxidant gene expression downstream of Nrf2: superoxide dismutase-1 (SOD1) (**C**), catalase (**D**), and thioredoxin (TRDX) (**E**). Values are presented as mean±SEM of 6 experiments in triplicate and represent the mRNA expression relative to GAPDH. *P<0.05 vs vehicle control human pulmonary artery smooth muscle cells (hPASMCs); ‡*P*<0.05 vs vehicle (V) pulmonary arterial hypertension (PAH)-hPASMCs determined by ANOVA with Tukey post hoc test.

16αOHE1 had no significant effect on Nrf2 in control hPASMCs but reduced Nrf2 activity in PAH-hPASMCs. This effect seems to be dependent on conversion of estrogen to 16αOHE1 as inhibition of CYP1B1, by 2,3′,4,5′-tetramethoxystilbene, normalized Nrf2 activity in PAH-hPASMCs (Figure 3A). Expression of Bach1 (BTB and CNC homology 1), a Nrf2 transcriptional repressor, was increased by 16αOHE1 (Figure 3B) in control hPASMCs at 2, 8, and 48 hours of stimulation. Basal levels of Nrf2-regulated antioxidants, SOD1, catalase, and thioredoxin, were decreased in PAH-hPASMCs compared with control hPASMCs. 16αOHE1 did not further modulate thioredoxin transcript levels. However, 16αOHE1 further reduced SOD1 in control hPASMCs where catalase was further reduced by 16αOHE1 in PAH-hPASMCs (Figure 3C and 3E).

### 16αOHE1 Influences Redox Signaling

**Figure 4.**
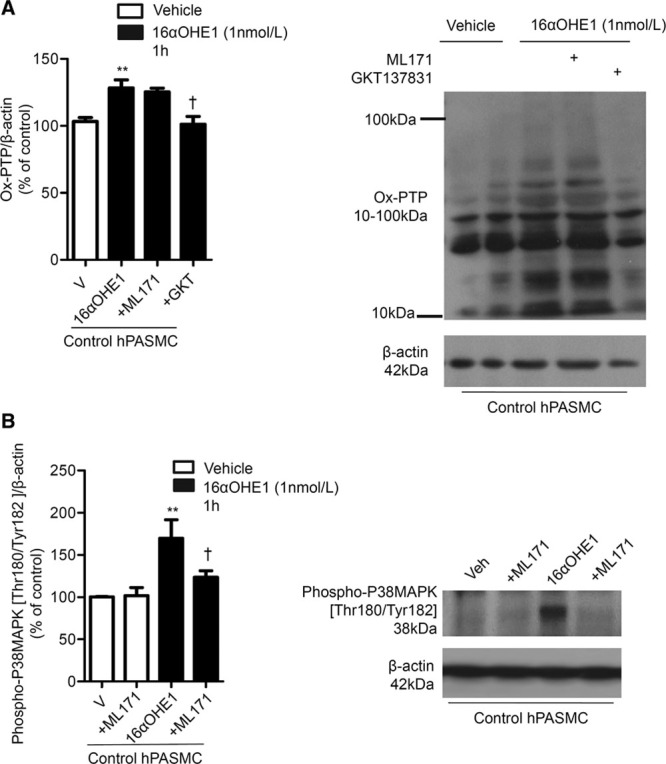
16α-hydroxyestrone (16αOHE1)–induced phosphorylation of p38MAPK and oxidation of protein tyrosine phosphatases (PTP). Irreversible oxidation of PTPs using the oxPTP antibody (**A**) and p38mitogen-activated protein kinase (p38MAPK) phosphorylation (**B**) were assessed after 16αOHE1 stimulation (1 h) in the presence or absence of ML171 and GKT137831. Results are representative of 5 experiments where protein expression is relative to β-actin. **P<0.01 vs vehicle control human pulmonary artery smooth muscle cells (hPASMCs); †*P*<0.05 vs 16αOHE1-treated control hPASMCs, determined by ANOVA with Tukey post hoc test.

One of the most important consequences of oxidative stress is oxidation of proteins, particularly redox-sensitive PTPs, which regulate phosphorylation of downstream proteins, including mitogen-activated protein kinases, such as p38mitogen-activated protein kinase. 16αOHE1 significantly increased irreversible PTP oxidation in hPASMCs, an effect that was inhibited by GKT137831 (Figure 4A). 16αOHE1 induced a significant increase in phosphorylation of p38mitogen-activated protein kinase in hPASMCs, an effect that was attenuated in control hPASMCs pretreated with Nox1 inhibitor, ML171 (Figure 4B).

### 16αOHE1-Induced Proliferation Involves Nox

**Figure 5.**
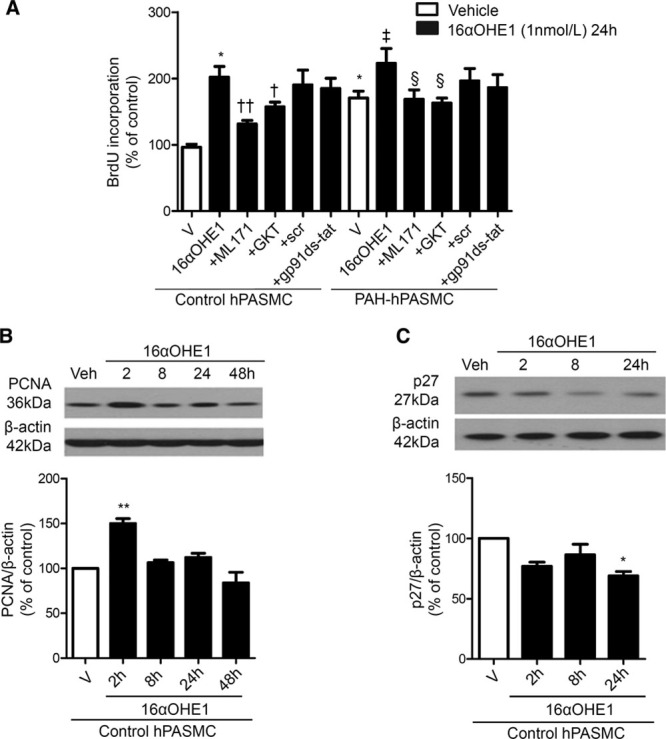
Role of nicotinamide adenine dinucleotide phosphate oxidase (Nox) in 16α-hydroxyestrone (16αOHE1)–mediated cell proliferation. To test if 16αOHE1 regulates proliferation in a Nox/reactive oxygen species–dependent manner, 5-bromo-2′-deoxyuridine (BrdU) incorporation was assessed in control human pulmonary artery smooth muscle cells (hPASMCs) and pulmonary arterial hypertension (PAH)-hPASMCs cultured for 24 h in the presence or absence of ML171, GKT137831, or gp91ds-tat (**A**). Results are representative of 6 experiments, in triplicate, where data are expressed relative to vehicle control conditions. Protein expression of cell growth marker proliferating cell nuclear antigen (PCNA) in response to 16αOHE1 (**B**) and cyclin-dependent kinase p27 (**C**) were determined by immunoblotting. Results are expressed as mean±SEM of 5 experiments where protein expression is relative to β-actin. *P<0.05 and **P<0.01 vs vehicle control hPASMCs; †*P*<0.05 and ††*P*<0.01 vs 16αOHE1-treated control hPASMCs; ‡*P*<0.05 vs vehicle PAH-hPASMCs; §*P*<0.05 vs 16αOHE1-treated PAH-hPASMCs determined by ANOVA with Tukey post hoc test.

16αOHE1 stimulated cell growth, as measured by 5-bromo-2′-deoxyuridine incorporation in control hPASMCs and PAH-hPASMCs (Figure 5A). These effects were attenuated by GKT137831 and ML171 but not by gp91ds-tat. In addition, expression of DNA polymerase accessory factor and proliferation marker proliferating cell nuclear antigen was increased by 16αOHE1 at 2 hours (Figure 5B). Cyclin-dependent kinase inhibitor, p27, is able to bind to a broad spectrum of cyclin/cyclin-dependent kinase complexes, inhibiting their activities, and as such can inhibit progression at every cell cycle phase. Therefore, decreased p27 levels have been associated with increased cell proliferation.^31^ Consistent with effects on proliferating cell nuclear antigen, 16αOHE1 decreased p27 protein expression at 24 hours of stimulation (Figure 5C).

### Effects of Estrogen and 16αOHE1 Are Specific to hPASMCs

To evaluate whether redox and proliferative effects of 16αOHE1 are generalized phenomena or specific for hPASMCs, we also studied hVSMCs from peripheral arteries. 16αOHE1 increased ^·^O_2_^−^ production in control hPASMCs but not in hVSMCs (Figure S4A). 16αOHE1 did not affect cell proliferation in hVSMCs (Figure S4B). Estrogen decreased ROS production in hVSMCs at 2 hours (Figure S5A). This had no effect on subsequent 5-bromo-2′-deoxyuridine incorporation in hVSMCs (Figure S5B).

### Genetic Ablation of Nox1 and Nox4 Attenuates Development of Pulmonary Hypertension in Female Mice

**Figure 6.**
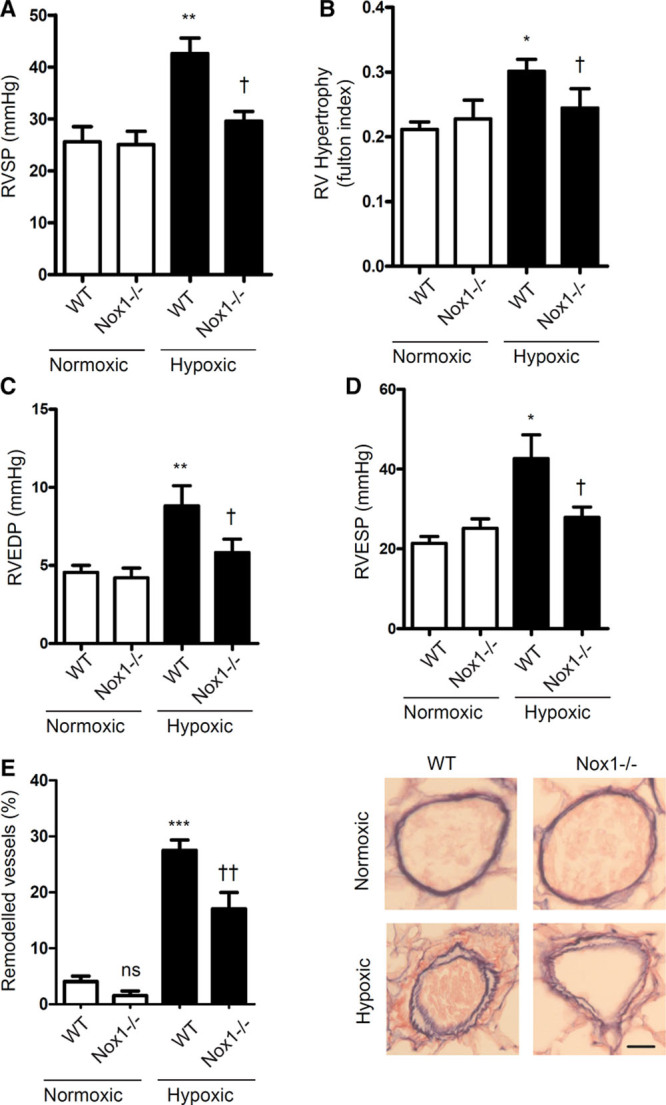
Effects of Nox1^−/−^ on hypoxia-induced pulmonary hypertension in female mice. Pressure–volume loop was used to assess hemodynamic parameters to evaluate the development of pulmonary hypertension in Nox1^−/−^ mice. Effects of Nox1^−/−^ on right ventricular (RV) systolic pressure (RVSP) (**A**) and RV hypertrophy, calculated as RV weight/left ventricular+septum weight (**B**). RV end-diastolic pressure (RVEDP) (**C**), RV end-systolic pressure (RVESP) (**D**). Percentage of pulmonary vascular remodeling in distal pulmonary arteries in normoxic and hypoxic mice with representative images of pulmonary arteries (elastin Van Gieson stain; scale bar, 50 µm) (**E**). Data are presented as mean±SEM; n=8 to 10 per group. *P<0.05, **P<0.01, and ***P<0.001 vs wild-type (WT) normoxic; †*P*<0.05 and ††*P*<0.01 vs WT hypoxic, determined by 2-way ANOVA with Tukey post hoc test. ns indicates not significant.

To evaluate the pathophysiological significance of our in vitro findings, we extended our studies to an estrogen-dependent mouse model of pulmonary hypertension by examining female Nox1^−/−^ and Nox4^−/−^ mice exposed to hypoxic conditions. Under normoxic conditions, RVSP and RVH were not different between WT and Nox1^−/−^ mice. Under hypoxic conditions, RVSP, RVH, RV end-diastolic pressure, and RV end-systolic pressure (Figure 6A through 6D) were increased in WT controls, responses that were attenuated in hypoxic Nox1^−/−^ mice (Figure 6). Similarly, vascular thickening (remodeling), as assessed by histological analysis, was increased in hypoxic compared with normoxic WT mice. No significant effects on vascular remodeling were observed in normoxic WT versus Nox1^−/−^ mice. However, under hypoxic conditions, Nox1^−/−^ mice exhibited reduced vascular remodeling compared with hypoxic WT mice (Figure 6E). Cardiac output was decreased in hypoxic WT compared with normoxic WT mice (Figure S6A). Mean arterial pressure, stroke volume, left ventricular systolic pressure, left ventricular hypertrophy, and heart rate were unchanged across experimental groups (Figure S6B through S6F).

**Figure 7.**
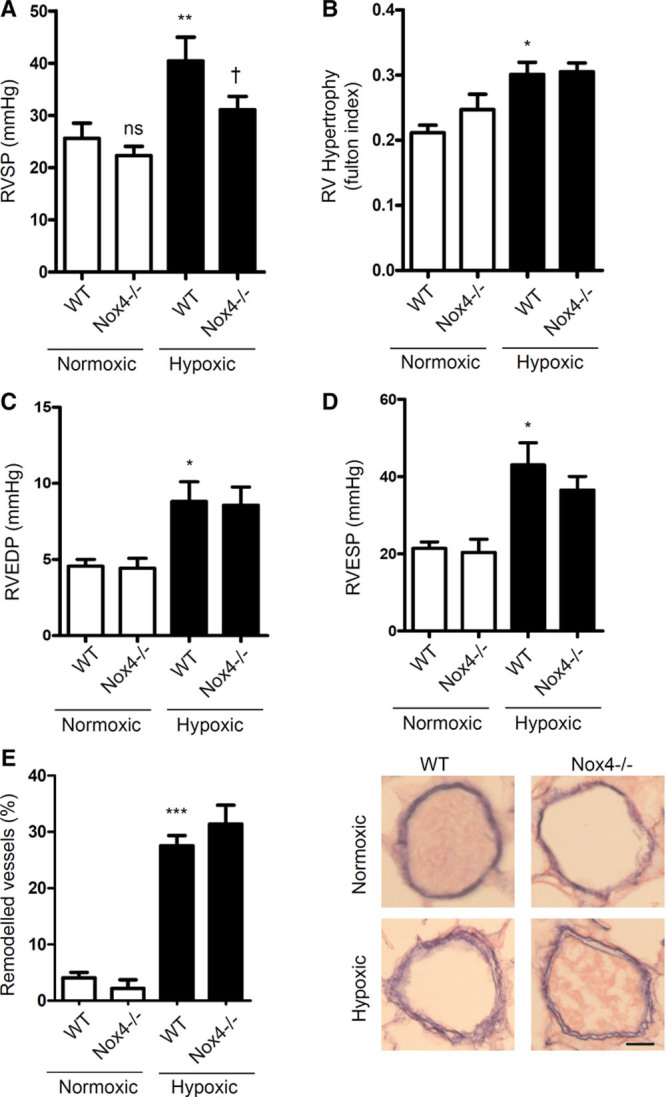
Effects of Nox4^−/−^ on hypoxia-induced pulmonary hypertension in female mice. Pressure–volume loop was used to assess hemodynamic parameters to evaluate development of pulmonary hypertension female Nox4^−/−^ mice. Effects of Nox4^−/−^ on right ventricular (RV) systolic pressure (RVSP) (**A**) and RV hypertrophy, calculated as RV weight/left ventricular+septum weight (**B**). RV end-diastolic pressure (RVEDP) (**C**) and RV end-systolic pressure (RVESP) (**D**). Percentage of pulmonary vascular remodeling in distal pulmonary arteries in normoxic and hypoxic mice with representative images of pulmonary arteries (Elastin Van Giesen stain; scale bar, 50 µm) (**E**). Data are presented mean±SEM; n=8 to 10 per group. *P<0.05, **P<0.01, and ***P<0.001 vs wild-type (WT) normoxic; †*P*<0.05 vs WT hypoxic, determined by 2-way ANOVA with Tukey post hoc test. ns indicates not significant.

As shown in Figure 7A, in WT mice, hypoxia increased RVSP, and this was attenuated in hypoxic Nox4^−/−^ mice (Figure 7A). Hypoxic WT mice also showed increases in RVH, RV end-diastolic pressure, RV end-systolic pressure, pulmonary vascular remodeling (Figure 7B through 7E); with decreased cardiac output (Figure S7A), hallmarks of pulmonary hypertension. However, these effects remained similar in Nox4^−/−^ mice. No changes in mean arterial pressure, stroke volume, left ventricular systolic pressure, left ventricular hypertrophy, or heart rate were observed across the study groups (Figure S7B through S7F).

### Expression of CYP1B1 in Female Mice With Pulmonary Hypertension

To evaluate indirectly whether estrogen metabolism in pulmonary arteries may be altered in mice deficient in Nox, we assessed expression of CYP1B1, which catalyses estrogen to its metabolites, in pulmonary arteries of normoxic and hypoxic WT Nox1^−/−^ and Nox4^−/−^ mice. Expression of CYP1B1, at the protein and mRNA levels, was increased in hypoxic WT and Nox4^−/−^ mice but not in hypoxic Nox1^−/−^ mice (Figure S8).

## Discussion

**Figure 8.**
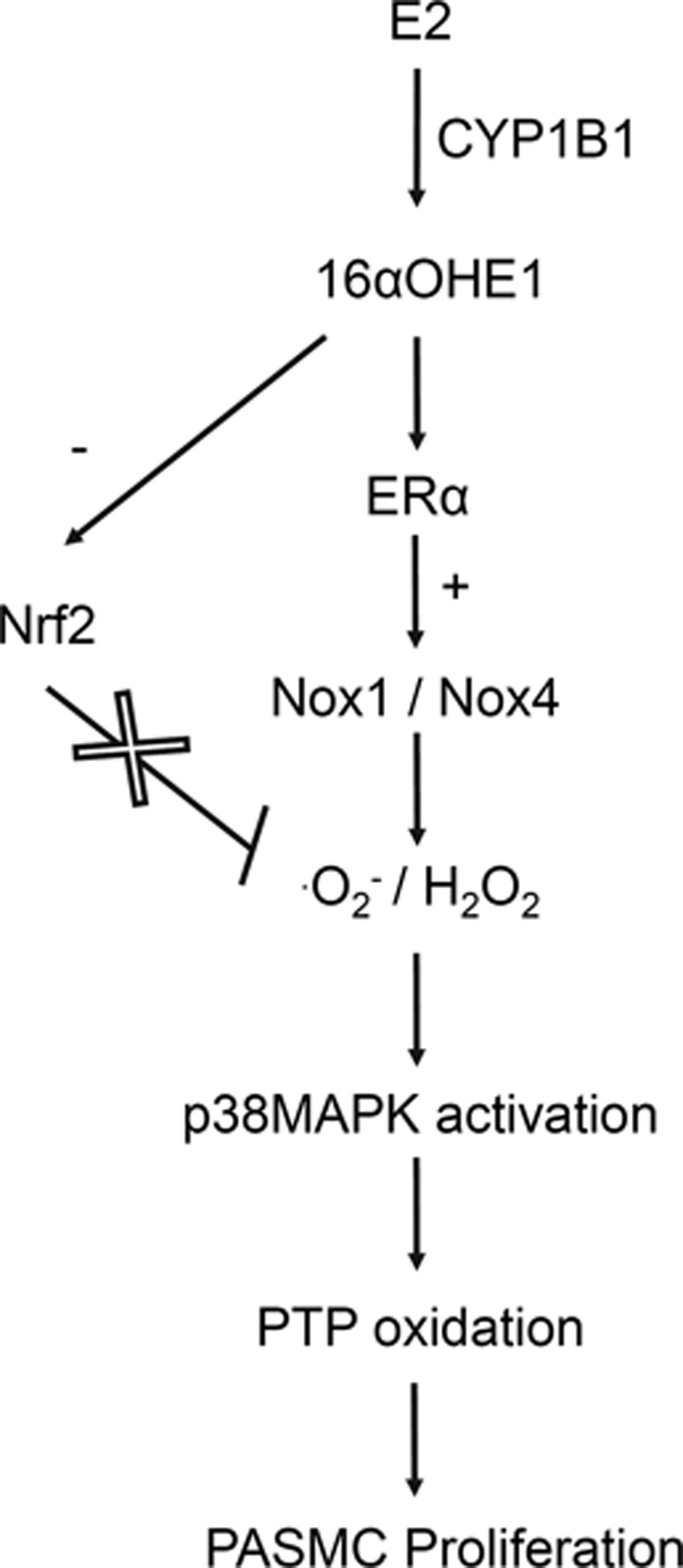
Schematic of putative role of 16α-hydroxyestrone (16αOHE1) in human pulmonary artery smooth muscle cells (PASMCs). The actions of 16αOHE1 and estrogen (E2) are mediated not only via the E2 receptors but also involve the activation of nicotinamide adenine dinucleotide phosphate oxidases (Noxs), which leads to ^·^O_2_^−^ and hydrogen peroxide (H_2_O_2_) production. Excessive reactive oxygen species production coupled with impaired antioxidant mechanisms in response to 16αOHE1 may promote oxidation of protein tyrosine phosphatases (PTP) and enhanced signaling through p38mitogen-activated protein kinase (p38MAPK) and proteins involved in cell cycle regulation, leading to deleterious oxidative stress and pulmonary vascular proliferation. + indicates activation; and −, inhibition.

Major findings from our study show that in PASMCs from female subjects, the estrogen metabolite 16αOHE1 induces ROS production, downregulates the protective antioxidant effects of Nrf2, stimulates redox signaling, and promotes cell growth (Figure 8). Processes underlying these actions involve primarily Nox1 and ERα. 16αOHE1 effects are amplified in hPASMCs from female patients with PAH. In support of the importance of Nox1 in vascular processes associated with pulmonary hypertension, our in vivo studies showed that hypoxia-induced pulmonary hypertension and arterial remodeling were ameliorated in Nox1^−/−^ mice but not in Nox4^−/−^ mice. Nox1^−/−^ mice also had reduced pulmonary artery content of the estrogen-metabolizing enzyme CYP1B1. Our study provides new molecular insights through Nox1/ROS and Nrf2 whereby 16αOHE1 influences hPASMC function, which when upregulated may contribute to vascular injury and remodeling in PAH, particularly important in women.

Studies have linked hormone replacement therapy and the contraceptive pill to the increased incidence of PAH^32^ giving rise to the hypothesis that estrogen and its metabolites play a role in the pathobiology of PAH. A metabolic shift toward the formation of proproliferative estrogen metabolites, including 16αOHE1, by altered expression of CYP1B1, is associated with the development and progression of PAH.^1^ Although CYP1B1 expression is low under basal conditions, expression is upregulated in PAH. Relatedly, inhibition or loss-of-function of CYP1B1 is protective in preclinical PAH models, thus demonstrating that CYP1B1 is involved in the pathogenesis of PAH.^1^ In support of this, a CYP1B1 single nucleotide polymorphism has been associated with PAH and oncogenesis, and these pathways are thought to underpin sexual dimorphism in RV failure.^12^ In addition, 16αOHE1 upregulates MicroRNA-29, which alters molecular and functional indices of energy metabolism, contributing to PAH.^33^ Other pathways, involving ROS, have also been suggested in PAH and seem to be regulated by 16αOHE1 as we demonstrate here.

The ability of estrogen to induce ROS in hPASMCs was dependent on CYP1B1 activity and likely the production of estrogen metabolites because pharmacological inhibition of CYP1B1 prevented estrogen-induced, but not 16αOHE1-induced, ROS production. Estrogens exert their cellular actions by activating their receptors, ERα, ERβ, and G-protein–coupled ER.^34^ In PAH, opposing roles of ERα and ERβ are described where increased pulmonary ERα expression has been associated with proliferation of PASMCs in human and experimental PAH.^35,36^ Inhibition of ERα reverses PAH in female mice but not in male hypoxic mice.^35,36^ Protective actions of estrogen on cell proliferation are mediated predominantly via ERβ signaling.^37^ In our studies and in line with others, we observed that 16αOHE1-induced ROS generation is mediated through ERα because 16αOHE1-mediated effects were blocked by 1,3-bis(4-hydroxyphenyl)-4-methyl-5-[4-(2-piperidinylethoxy) phenol]-1*H*-pyrazole dihydrochloride (ERα antagonist) but not by 4-[2-phenyl-5,7-bis(trifluoromethyl)pyrazolo[1,5-*a*]pyrimidin-3-yl]phenol (ERβ antagonist).

Hydroxyestrogens have also been shown to induce DNA damage either directly, through formation of DNA adducts, or indirectly, through redox cycling and generation of ROS.^38^ This further strengthens the concept that the deleterious actions of estrogen may be dependent on the conversion to 16αOHE1 where results of our study implicated the generation of ROS as a mediator of these deleterious actions. Exact mechanisms of ROS production, especially with respect to estrogen/16αOHE1, are poorly understood. However, growing evidence implicates a role for Noxs, particularly Nox1 and Nox4, in the development and progression of PAH.^39,40^ We found increased basal levels of Nox1 and Nox4 in PAH-hPASMCs compared with control hPASMCs in agreement with other studies.^18,40^ 16αOHE1 induced an increase in expression of Nox1 and Nox subunits associated with Nox1 activation, whereas Nox4 and its regulator poldip2 were not significantly modified in PAH-hPASMCs. These findings emphasize the preferential importance of Nox1 versus Nox4 in PAH. Despite a role for Nox2 being reported by Liu et al,^41^ we were unable to delineate a role for Nox2 in our study. This may be related to the fact that Nox2 was almost undetectable in hPASMCs in our study.

Although Nox1 primarily produces ^·^O_2_^−^ and is reported to have deleterious effects in various components of the cardiovascular system, a protective role of Nox4, which primarily produces H_2_O_2_, has been suggested.^42^ As such, Nox1 and Nox4 may exert opposing effects within the same tissue because of the difference in the reactive species produced. In our study, basal levels of H_2_O_2_ in PAH-hPASMCs were reduced, whereas 16αOHE1 was associated with excessive production of H_2_O_2_ in PAH-hPASMCs. It is well established that high concentrations of ROS can trigger the oxidation of downstream signaling molecules, such as PTPs, resulting in the loss of function as a phosphate acceptor.^43^ In association with excessive ROS production by 16αOHE1, we found an increase in irreversibly oxidized PTPs and enhanced phosphorylation of p38mitogen-activated protein kinase, which is downstream of PTP. Our findings support the notion that PTP inhibition is important in PAH, and we suggest that 16αOHE1-induced ROS may be important in this process.

Our data indicate that in addition to regulating ROS production, 16αOHE1 influences antioxidant systems in hPASMCs. In PAH-hPASMCs, 16αOHE1 increased ROS production and decreased Nrf2 activation, suggesting overall ROS accumulation and oxidative stress. This effect was dependent on CYP1B1, suggesting a role for estrogen to 16αOHE1 conversion in Nrf2 dysfunction in PAH-hPASMCs. Previous studies have reported Nox-derived ROS activating Nrf2 nuclear translocation in physiological states; however, in conditions of oxidative stress and systemic vascular pathology, Nrf2 activation by Nox-derived ROS is dysregulated.^19^ Our data suggest that similar dysregulation of Nrf2 may occur in pathophysiological conditions of the pulmonary vasculature.

In addition, we observed a decrease in the H_2_O_2_-reducing enzymes, catalase, and thioredoxin in PAH-hPASMCs compared with control hPASMCs, where effects on catalase were further reduced in 16αOHE1-treated PAH-hPASMCs. This may be indicative of increased production and accumulation of H_2_O_2_ in PAH, potentiated by 16αOHE1. To better understand the functional significance of 16αOHE1-induced oxidative stress, we studied effects on proliferation, a hallmark of pulmonary vascular remodeling. 16αOHE1 stimulated ROS production in hPASMCs, expression of proliferating cell nuclear antigen and cell cycle inhibitors, and increased proliferation, effects that were blocked by inhibitors of Nox1.

Taken together, our cell-based studies demonstrate that in hPASMCs, 16αOHE1 stimulates ROS production through ERα–Nox–dependent processes. In PAH-increased Nox1 expression, oxidative stress and downregulation of the antioxidant Nrf2 system lead to increased PTP oxidation, aberrant redox signaling, and cell proliferation, as summarized in Figure 8. Because hPASMCs were derived from younger women than the patient cohort from whom we obtained PAH-hPASMCs cells, we cannot exclude the possibility that aging or menopausal state may play a role. However, the differences observed in our study are likely independent of changes in ER status because vascular protein expression of ER does not change significantly with aging.^44^ The cellular phenomena observed in our study seem to be specific for hPASMCs because hVSMCs from other vascular beds failed to respond to estrogen and 16αOHE1.

To further test the pathophysiological significance of our findings, we performed in vivo studies in Nox1^−/−^ and Nox4^−/−^ mice exposed to hypoxia. WT mice exhibited features of pulmonary hypertension when exposed to hypoxic conditions as evidenced by increased RVSP, RV end-systolic pressure, RV end-diastolic pressure and RVH, processes that were attenuated in Nox1^−/−^ mice. Associated with pulmonary hypertensive changes, pulmonary arteries displayed significant remodeling, which was reduced in Nox1^−/−^ mice but not in Nox4^−/−^ mice. A role for 16αOHE1 may be implicated here through the absence of hypoxia-induced increases in CYP1B1 expression in Nox1^−/−^ mice. Only modest protective effects on RVSP were evident in hypoxic PH Nox4^−/−^ mice; this is in alignment with a previous study using a small-molecule Nox4 inhibitor, showing some effects on RVH and vascular remodeling.^45^ As such, the important role for Nox1 in mediating PAH phenotypes that were observed in vitro were paralleled in vivo where Nox1^−/−^ mice were protected from hypoxia-induced vascular injury.

In conclusion, using hPASMCs from patients we show that the estrogen metabolite 16αOHE1 increases Nox-dependent ROS generation and decreases Nrf2–antioxidant systems that contribute to oxidative damage and redox-sensitive proliferation of hPASMCs (represented schematically in Figure 8), processes critically involved in PAH. We identify Nox1 as being particularly important in hypoxia-induced pulmonary hypertension and in 16αOHE1-mediated vascular effects in PAH. Our study provides new molecular insights through Nox1/ROS and Nrf2 whereby 16αOHE1 influences pulmonary artery VSMC function, which when upregulated may contribute to vascular injury and remodeling in PAH. Such phenomena may be especially important in female mice.

### Perspectives

Metabolites of estrogen, including 16αOHE1, participate in many physiological processes implicated in cardiovascular complications associated with PAH. Current advances in the understanding of estrogen metabolism have provided insights into mechanisms involved in cardiopulmonary diseases. Our results identify Nox-dependent redox signaling of 16αOHE1 as an important player in the molecular and cellular processes associated with PAH. Nox1 is identified as a major player in 16αOHE1 effects.

## Acknowledgments

We thank Professor C. Yabe-Nishimura (Kyoto Prefectural University of Medicine, Japan) for providing Nox1^−/−^ mice and Dr K. Schröder (Goethe University of Frankfurt, Germany) for providing Nox4^−/−^ mice. We are grateful to Professor Nicholas W. Morrell (University of Cambridge, United Kingdom) for the supply of human pulmonary artery smooth muscle cells. Dr Anne Katrine Johansen (Hubrecht Institute, The Netherlands) for assistance with experimental design and Carol Jenkins for the technical support. We thank Genkyotex for supplying GKT137831.

## Sources of Funding

This study was funded by grants from the British Heart Foundation (R.M. Touyz: CH/12/4/29762 and RG/13/7/30099 and M.R. MacLean: RG/11/7/28916). K.Y. Hood was supported by a PhD studentship from Biotechnology and Biological Sciences Research Council (2012/168760-01).

## Disclosures

None.

## Supplementary Material

## Novelty and Significance

### What Is New?

- This study demonstrates that 16α-hydroxyestrone plays an important role in molecular and cellular processes associated with vascular injury and dysfunction through reactive oxygen species generation in pulmonary hypertension.

### What Is Relevant?

- Female mice develop pulmonary arterial hypertension more frequently than male mice. Estrogen metabolites, such as 16α-hydroxyestrone, and alterations in estrogen metabolism have also been implicated in the pathobiology of pulmonary arterial hypertension.
- 16α-hydroxyestrone stimulates nicotinamide adenine dinucleotide phosphate oxidase–derived reactive oxygen species generation, downregulates nuclear factor estrogen–related factor 2 and antioxidant systems, and induces proliferation, effects that are specific to pulmonary artery smooth muscle cells. 16α-hydroxyestrone stimulates pulmonary artery smooth muscle cell reactive oxygen species production through estrogen receptor-α.
- These data may contribute to a better understanding of cardiovascular complications in conditions associated with altered metabolism of estrogen, such as in pulmonary hypertension.

### Summary

Increased levels of 16α-hydroxyestrone production have been implicated in pulmonary arterial hypertension, yet the underlying mechanisms remain elusive. Here, we describe a mechanism of action for 16α-hydroxyestrone and suggest that conversion of estrogen to 16α-hydroxyestrone leads to increased nicotinamide adenine dinucleotide phosphate oxidase-1– and nicotinamide adenine dinucleotide phosphate oxidase-4–dependent reactive oxygen species production, disrupted redox-sensitive protein tyrosine phosphatase and mitogen-activated protein kinase signaling, and decreased antioxidants, which contribute to oxidative damage and proliferation of pulmonary artery smooth muscle cells.
