# Supplementary material for: Nicotinamide Adenine Dinucleotide Phosphate Oxidase–Mediated Redox Signaling and Vascular Remodeling by 16α-Hydroxyestrone in Human Pulmonary Artery Cells: Implications in Pulmonary Arterial Hypertension
Source: Hypertension. 2016 Aug 10;68(3):796–808. doi: 10.1161/HYPERTENSIONAHA.116.07668 (PMC4978604; doi:10.1161/HYPERTENSIONAHA.116.07668)
Supplement: Supplementary file 1 [file hyp-68-796-s001.pdf]

**On-line Data Supplement**

# Nox-mediated redox signaling and vascular remodeling by 16α-hydroxyestrone in human pulmonary artery cells: Implications in pulmonary arterial hypertension.

Katie Y Hood BSc, Augusto C Montezano PhD, Adam P Harvey PhD, Margaret Nilsen, Margaret R MacLean* PhD, Rhian M Touyz* MD PhD.
Institute of Cardiovascular and Medical Sciences, University of Glasgow, UK.
*Joint senior authors

**Short title:** Estrogen, Nox and pulmonary hypertension.

**Address correspondence to:**
Rhian M. Touyz MD, PhD
Institute of Cardiovascular and Medical Sciences,
BHF Glasgow Cardiovascular Research Centre,
126 University Place, University of Glasgow, G12 8TA, UK.
Tel: +44(0)141 330 7775; Fax: +44(0)141 330 3360
Email: rhian.touyz@glasgow.ac.uk

# Detailed Methods

## Reagents

The following antibodies were used: anti-Bach1 (Santa Cruz, sc-14700); anti-PCNA (Santa Cruz, sc-56); anti-p27(Santa Cruz, sc-1641); anti-Nox1 (Abcam, ab55831); anti-Nox4 (Abcam, ab133303); anti-CYP1B1 (Abcam, ab78044); anti-phospho-P38MAPK (Cell signalling, 9211s); anti-Ox-PTP (R&D systems, MAB2844) and anti-β-actin (Abcam, ab8229).

E2 (Cat.#2824) was purchased from Tocris, UK. 16αOHE1 (Cat.#E1250-000) was purchased from Steraloids, Newport, USA. MPP, an estrogen receptor alpha (ERα) antagonist (Cat.#M7068) [1] and PHTPP, an estrogen receptor beta (ERβ) antagonist (Cat.#SML1355) were purchased from Sigma, UK [1]. GKT137831, a Nox1/4 inhibitor, was gifted by Genkyotex, Switzerland [2]. ML171, a Nox1 inhibitor (Cat.#4653); SOD mimetic, tempol (Cat.#3082) [3] and Cytochrome P450 1B1 inhibitor, 2,3',4,5'-Tetramethoxystilbene (TMS; Cat.#1509) [4] were purchased from Tocris, UK. Gp91ds-tat (Cat#AS-63818) [5], a peptide Nox2 inhibitor and scrambled control peptide were purchased from AnaSpec, USA.

## Cell culture

Cells were cultured in Dulbecco's Modified Eagle Medium (DMEM; Gibco, Paisley, UK) supplemented with antibiotic antimycotic solution (containing 0.25µg/ml amphotericin B; 100U/ml penicillin; 100µg/ml streptomycin; Sigma-Aldrich, Poole, UK) and 10% (v/v) fetal bovine serum (Sera Laboratories International, West Sussex, UK). Cells were grown to 70% confluence before serum deprivation for 24hours in charcoal-stripped 0.5% fetal bovine serum in phenol-red free DMEM prior to experiments to render them quiescent. Details of hPASMCs utilised can be found in Table S1.

## Lucigenin-enhanced chemiluminescence

ROS generation was assessed in cell lysates by lucigenin-enhanced chemiluminescence assay as previously described[3, 6]. Control hPASMC, PAH-hPASMCs and hVSMCs were stimulated with E2 or 16αOHE1 for 5 minutes to 48 hours. In some experiments, cells were pre-exposed for 30 minutes to ML171, GKT137831, gp91ds-tat or scrambled peptide, tempol, MPP or PHTPP. Inhibitor studies were carried out at the peak time points for ROS production, 4hours stimulation for E2 and 30 minutes for 16αOHE1 treatment, respectively. Cells were washed with ice-cold PBS and harvested in lysis buffer (20mmol/L of $KH_2PO_4$, 1 mmol/L of EGTA, 1µg/mL of aprotinin, 1µg/mL of leupeptin, 1µg/mL of pepstatin, and 1mmol/L of PMSF). 50µl of sample was added to a suspension containing 175µl of assay buffer (50mmol/L of $KH_2PO_4$, 1mmol/L of EGTA, and 150mmol/L of sucrose) and lucigenin (5µmol/L). Luminescence was measured with a luminometer (AutoLumat LB 953, Berthold) before and after stimulation with NADPH (100µmol/l). A buffer blank was subtracted from each reading. Superoxide anion production was inhibited by tempol and was expressed as relative luminescence units (RLU)/µg protein, relative to vehicle control conditions.

## Amplex Red assay

Hydrogen peroxide ($H_2O_2$) was assessed in cell lysates using the fluorescence assay Amplex Red Hydrogen Peroxide/Peroxidase Assay Kit (Life Technologies, Carlsbad, CA, USA) in accordance with manufacturer's instructions and as we have previously described[2]. Briefly, Amplex Red (50 µmol/L) and horseradish peroxidase (0.1 U/mL) were added to the cellular samples. Fluorescence readings were made in a 96-well plate at Ex/Em = 530/590 nm using 50 µl samples harvested in protein lysis buffer. $H_2O_2$ production was normalized to protein concentration. The results are expressed in arbitrary units per micro-gram protein, relative to vehicle control conditions.

## Immunoblotting

Proteins were extracted from cells and mouse pulmonary artery and immunoblotting was performed as we previously described[7]. All antibodies were used at 1:1000 dilution unless otherwise stated. After incubation with HRP-conjugated secondary antibodies, signals were revealed by chemiluminescence (WestPico, Pierce), visualized by autoradiography, and quantified densitometrically with open-source software ImageJ. Anti-β-actin antibody (1:10,000) was used as protein loading control.

## Nuclear factor (erythroid-derived 2)-like 2 (Nrf2) activity assay

To assess nuclear accumulation of Nrf2, samples were prepared according to the manufacturer's protocol using a nuclear extract kit (Active Motif, Carlsbad, CA). Nuclear preparations (10μg) were used for the TransAM Nrf2 ELISA kit (Active Motif, Carlsbad, CA) to measure DNA binding of activated Nrf2 nuclear protein, as determined by absorbance at 450nm, following manufacturer's instructions and as previously described.

## DNA synthesis by BrdU incorporation

Cell proliferation by E2 and 16αOHE1 was measured by BrdU incorporation using a Proliferation Assay kit (Calbiochem, Darmstadt, Germany), according to the manufacturer's instructions. Cells were seeded onto a 96-well plate and starved overnight before stimulation with E2 or 16αOHE1 in the absence or presence of ML171, GKT137831 or gp91ds-tat. Cells were incubated with BrdU for 24 hours. Absorbance was obtained at dual wavelength (450nm and 595nm) with a spectrophotometer (Spectra Max; Molecular Devices, Sunnyvale, CA, USA). The results were normalized as percent of control vehicle conditions.

## Hypoxia-induced pulmonary hypertension in Nox1-/- and Nox4-/- mice

In studies involving the influence of the estrous cycle on non-reproductive functions, vaginal smear cytology is used to determine the cycle phases[8]. This was carried out prior to normoxic or hypoxic exposure to ensure this experimental model was commenced on the same cycle day for all mice.

## Supplemental References


1. Wright A, Ewart M, Mair K, Nilsen M, Dempsie Y, Loughlin L, Maclean M. Oestrogen receptor alpha in pulmonary hypertension. *Cardiovascular Research*. 2015;106:206-216.
2. Neves K, Cat A, Lopes R, Rios F, Anagnostopoulou A, Lobato N, de Oliveira A, Tostes R, Montezano A, Touyz R. Chemerin regulates crosstalk between adipocytes and vascular cells through nox. *Hypertension*. 2015;66:657-666.
3. Touyz R, Yao G, Viel E, Amiri F, Schiffrin E. Angiotensin ii and endothelin-1 regulate map kinases through different redox-dependent mechanisms in human vascular smooth muscle cells. *Journal of Hypertension*. 2004;22:1141-1149.
4. White K, Johansen AK, Nilsen M, Ciuclan L, Wallace E, Paton L, Campbell A, Morecroft I, Loughlin L, McClure JD, Thomas M, Mair KM, MacLean MR. Activity of the estrogen-metabolizing enzyme cytochrome p450 1b1 influences the development of pulmonary arterial hypertension. *Circulation*. 2012;126:1087-1098.
5. Rey FE, Cifuentes ME, Kiarash A, Quinn MT, Pagano PJ. Novel competitive inhibitor of nad(p)h oxidase assembly attenuates vascular o(2)(-) and systolic blood pressure in mice. *Circ Res*. 2001;89:408-414.
6. Chignalia A, Schuldt E, Camargo L, Montezano A, Callera G, Laurindo F, Lopes L, Avellar M, Carvalho M, Fortes Z, Touyz R, Tostes R. Testosterone induces vascular smooth muscle cell migration by nadph oxidase and c-src-dependent pathways. Hypertension. 2012;59:1263-U1478.
7. Callera G, Montezano A, Yogi A, Tostes R, He Y, Schiffrin E, Touyz R. C-src-dependent nongenomic signaling responses to aldosterone are increased in vascular myocytes from spontaneously hypertensive rats. Hypertension. 2005;46:1032-1038.
8. Caligioni CS. Assessing reproductive status/stages in mice. Curr Protoc Neurosci. 2009; Appendix 4: Appendix 4.

**Table S1. PAH Patient and non-PAH patient information.**

| Patient Group | Sex | Age | Disease Status |
|---|---|---|---|
| Non-PAH | Female | 57 | COPD |
| | | 58 | Mild emphysema |
| | | 59 | Squamous cell carcinoma |
| | | 64 | Mild emphysema |
| | | 64 | Lung carcinoma |
| | | 70 | Lobectomy |
| | | 71 | Adenocarcinoma |
| PAH | Female | 24 | IPAH |
| | | 30 | HPAH (R899X) |
| | | 33 | IPAH |
| | | 41 | HPAH (N903S) |
| | | 53 | IPAH |
| | | 39 | IPAH |

Pulmonary artery smooth muscle cell subject origin and characteristics. Known characteristics of subjects from whom cells were derived. HPAH, heritable pulmonary arterial hypertension (gene mutation in parenthesis); IPAH idiopathic pulmonary arterial hypertension.

**Table S2. Primers for qRT-PCR analysis.**

| Gene | Forward Primer | Reverse Primer |
|---|---|---|
| GAPDH | GAGTCAACGGATTTGGTCGT | TTGATTTTGGAGGGATCTCG |
| Nox1 | TCACCAATTCCCAGGATTGA | TGTGGTCTGCACACTGGAAT |
| Nox4 | TGCAGCAAGATACCGAGATG | GTGATCATGAGGAATAGCAC |
| p47phox | AGTCCTGACGAGACGGAAGA | TACATGGACGGGAAGTAGCC |
| p67phox | AAGCTGTTTGCCTGTGAGGT | CTGCTTCCAGACACACTCCA |
| p40phox | TCCTCCTCAGTCGGATCAAC | TGATGGTGCTGATGGTGTCT |
| NoxA1 | CATGATGCCAGGTCCCTAAT | CTCTGCTCCTGGTAGGCAGT |
| NoxO1 | GGTCCCCAGTTCTGTGCTT | CGGTCTGACGTTTCCAACAC |
| poldip2 | AGTCTCTCTGGCACCTTGGA | GAGAAGGGAGGAATCCGAAC |
| p22phox | AAGAGGAAGAAGGGCTCCAC | GAGAGCAGGAGATGCAGGAC |
| SOD1 | GAAGGTGTGGGGAAGCATTA | ACATTGCCCAAGTCTCCAAC |
| Catalase | CGTGCTGAATGAGGAACAGA | AGTCAGGGTGGACCTCAGTG |
| TRDX | GCCTTTCTTTCATTCCCTCTC | CCCACCTTTTGTCCCTTCTT |
| CYP1B1 | GCAGAAACTTCAACCCGATAA | GAGCGGGGCGGAGAGT |

Primers targeted to the above genes were designed using Primer 3 software online, and were used to assess gene expression.

**S1**

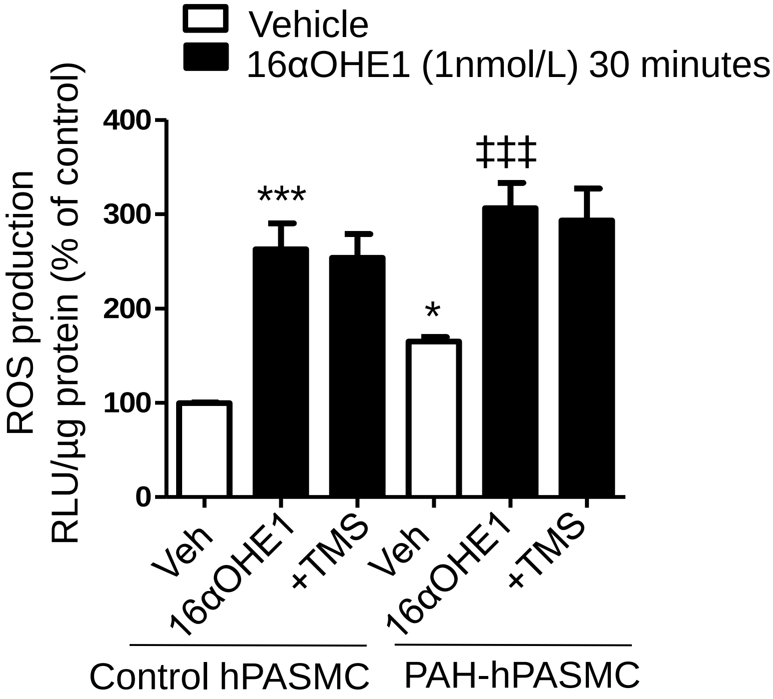


**S1: CYP1B1 inhibition by TMS has no effect on 16αOHE1-induced ROS formation.** Cells were exposed to TMS (100nmol/L) prior to 30 minutes stimulation with 16αOHE1 (A). Results are mean ±SEM of 6 experiments, in triplicate. *$p<0.05$; ***$p<0.001$ vs. vehicle control hPASMCs; ‡‡‡$p<0.001$ vs. vehicle PAH-hPASMCs, determined by ANOVA with Tukey's post-hoc test.

# S2

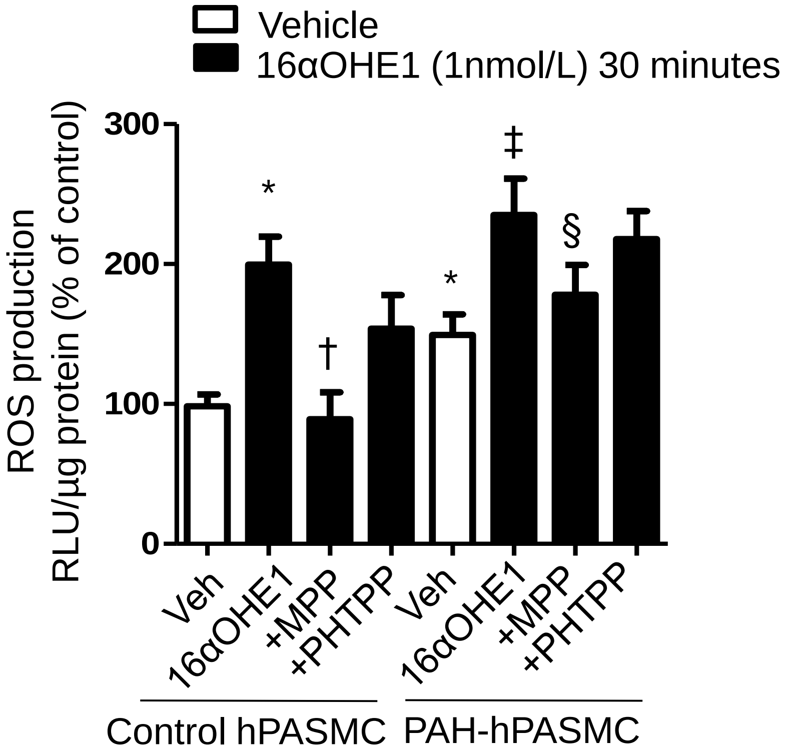


**S2: Contribution of the estrogen receptors to 16αOHE1-induced ROS production.** To investigate the role of the estrogen receptors in ROS production by 16αOHE1 cells were exposed to MPP (100nmol/L) and PHTPP (100nmol/L) (A). Results are mean ±SEM of 6 experiments, in triplicate. *$p<0.05$ vs. vehicle control hPASMCs; †$p<0.05$ vs. 16αOHE1-treated control hPASMCs; ‡$p<0.05$ vs. vehicle PAH-hPASMCs; §$p<0.05$ vs. 16αOHE1-treated PAH-hPASMCs, determined by ANOVA with Tukey’s post-hoc test.

**S3**

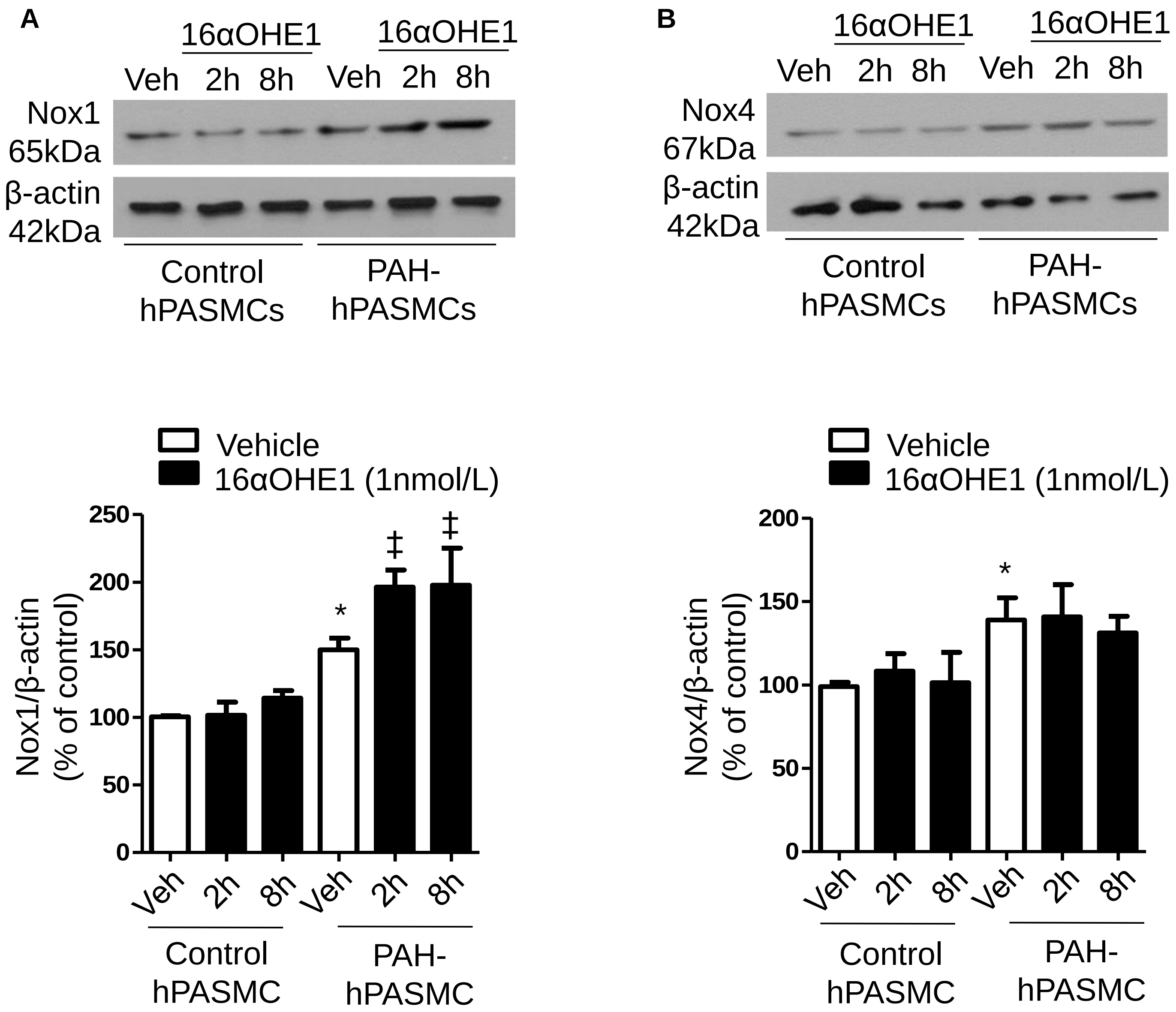


**S3: Effect of 16αOHE1 on NADPH oxidase isoform protein expression.** Protein expression levels of Nox1 (A) and Nox4 (B) assessed by Western blotting in response to 16αOHE1 at 2 and 8 hours in control hPASMC and PAH-hPASMCs. Results are mean ±SEM of 5 experiments, in triplicate. Graphs represent the protein expression relative to β-actin. *p<0.05 vs. vehicle control hPASMC; ‡p<0.05 vs. vehicle PAH-hPASMCs, determined by ANOVA with Tukey's post-hoc test.

# S4

**A**

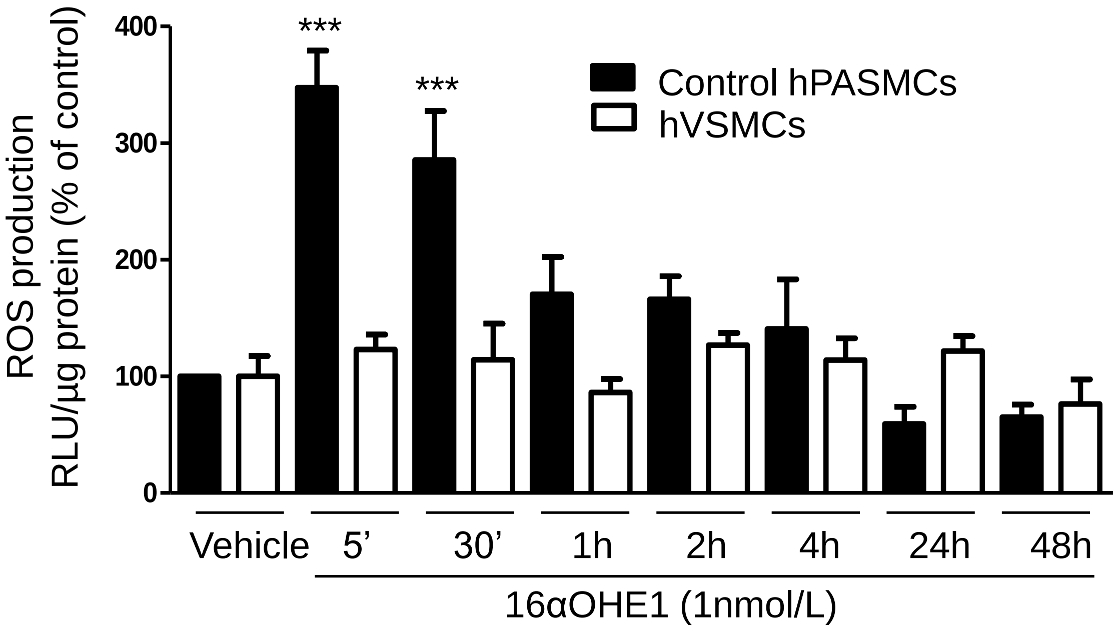


**B**

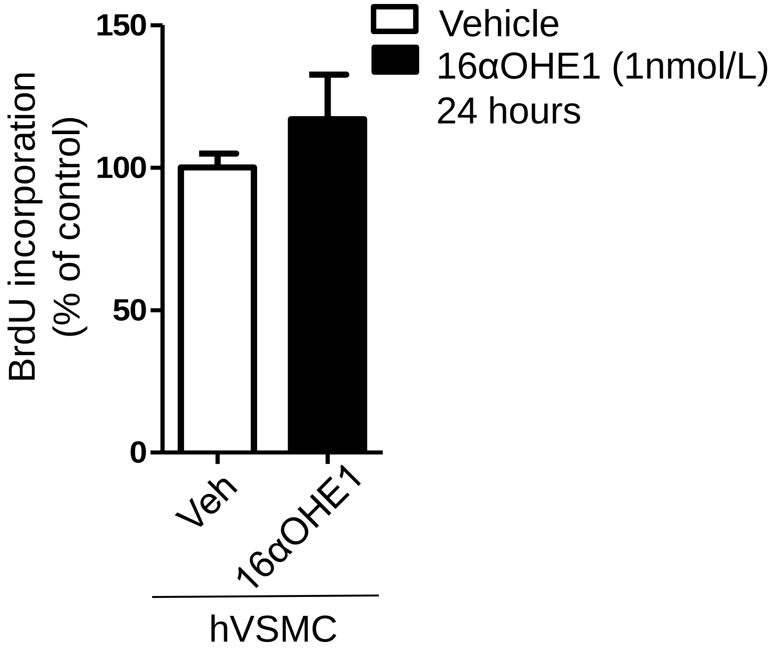


**S4: Effects of 16αOHE1 are pulmonary vascular bed-specific.** ROS production measured by lucigenin-enhanced chemiluminescence by 16αOHE1in control hPASMCs versus hVSMCs (A). Data are expressed as RLU/µg protein expressed as percentage of vehicle control conditions. BrdU incorporation by 16αOHE1 in hVSMCs (B). Results are mean ±SEM of 7 experiments, in triplicate. ***p<0.001 vs vehicle control hPASMCs determined by ANOVA with Tukey's post-hoc test

## S5

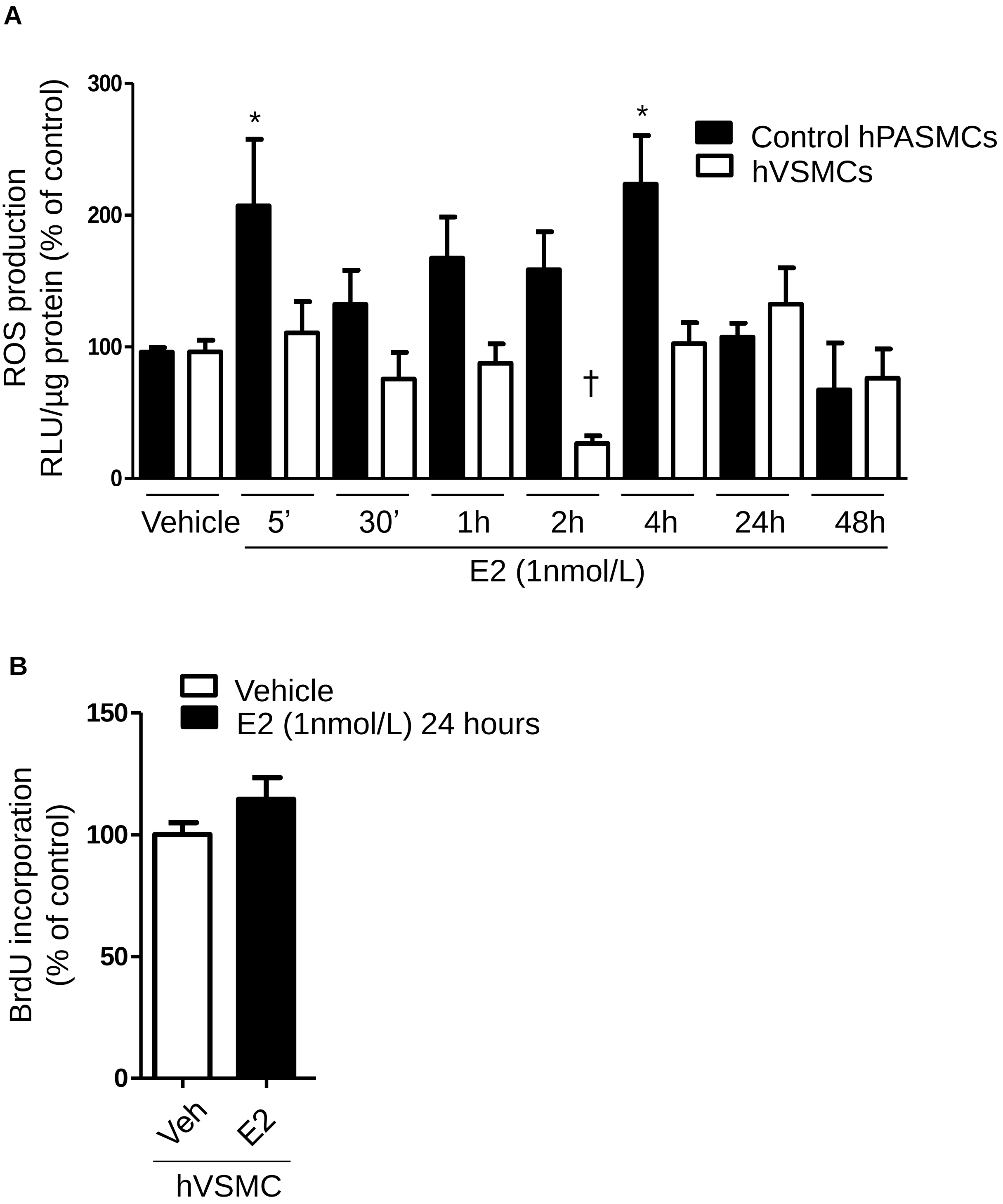


**S5: Effects of E2 are specific to the pulmonary vascular bed.** ROS production in cell lysates measured by lucigenin-enhanced chemiluminescence by E2 for 5 minutes to 48 hours in hPASMCs versus hVSMCs (A). Data are expressed as RLU/µg protein expressed as percentage of vehicle control conditions. Cell growth assessed by BrdU incorporation in hVSMCs by E2 (B), results are mean ±SEM of 6 experiments, in triplicate. Data are expressed as mean ±SEM. *p<0.05 vs. vehicle control hPASMCs; †p<0.05 vs. vehicle hVSMCs, determined by ANOVA with Tukey's post-hoc test.

# S6

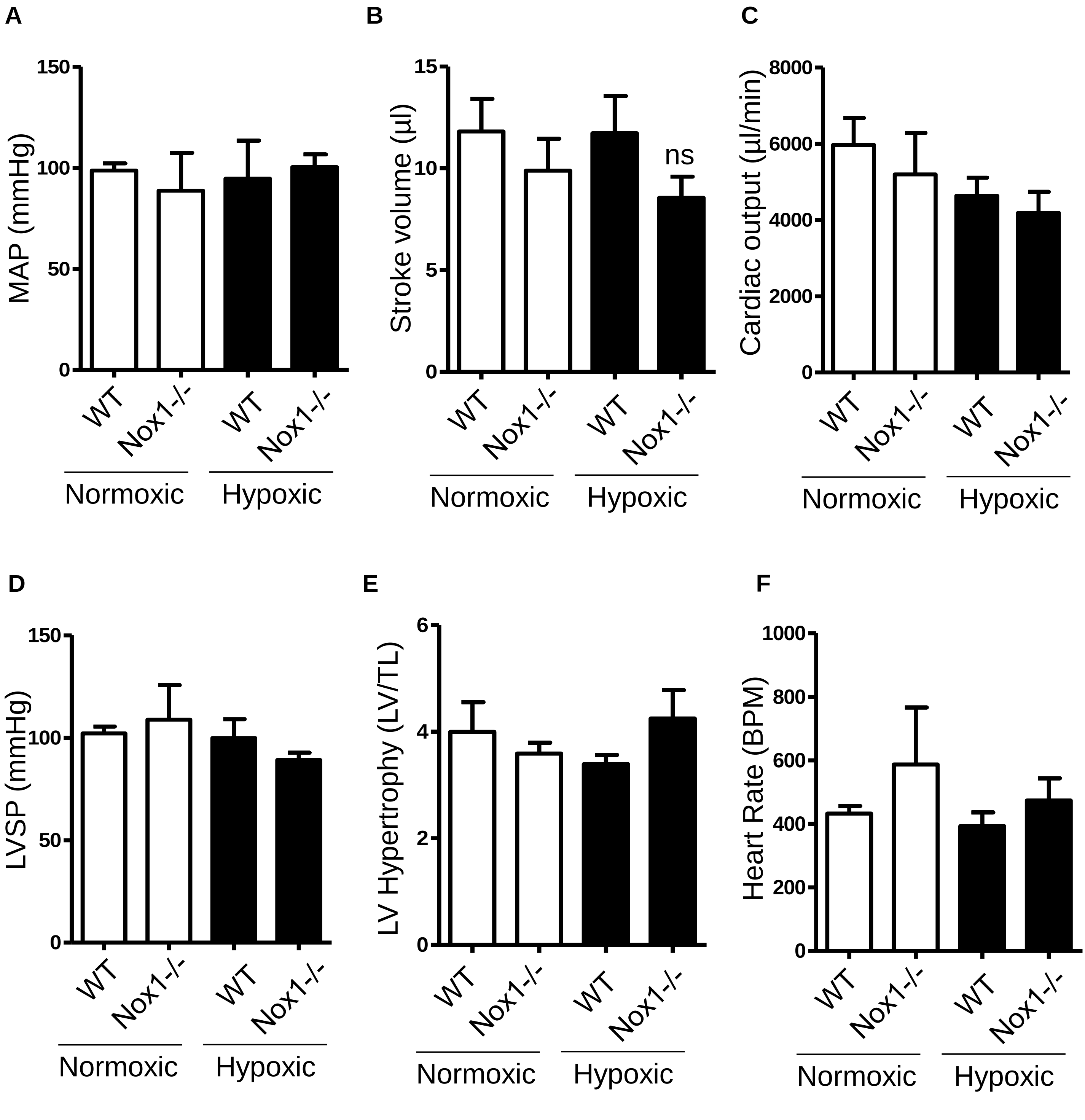


**S6: Pressure volume loop assessment of cardiac parameters in female WT and Nox1-/- mice**. Cardiac output (CO) (A), stroke volume (SV) (B), mean arterial pressure (MAP) (C), left ventricular systolic pressure (LVSP) (D), LV hypertrophy (LVH), relative to tibia length (E), heart rate (HR) (F). Results are mean ±SEM, n=8-10 per group. *$p<0.05$ vs WT normoxic, ns: not significant, determined by 2-way ANOVA with Tukey's post-hoc test. WT = wild-type, LV = left ventricle, TL = tibia length, BPM = beats per minute.

## S7

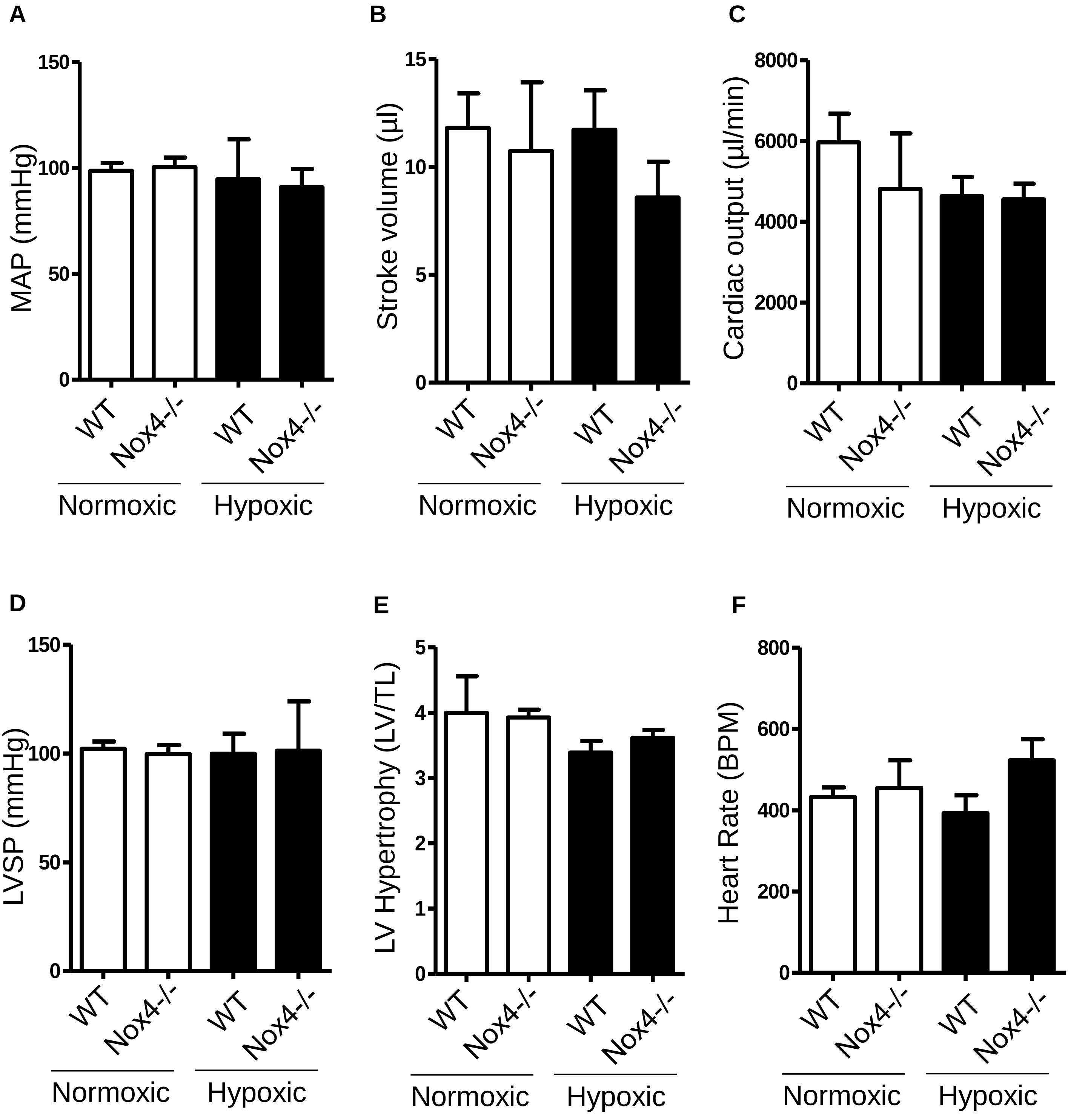


**S7: Pressure volume loop assessment of cardiac parameters in female WT and Nox4-/- mice**. Mean arterial pressure (MAP) (A), stroke volume (SV) (B), cardiac output (CO) (C), left ventricular systolic pressure (LVSP) (D), LV hypertrophy (LVH), relative to tibia length (E), heart rate (HR) (F). Results are mean ±SEM, n=8-10 per group. *p<0.05 vs WT normoxic, determined by 2-way ANOVA with Tukey's post-hoc test. WT = wild-type, LV = left ventricle, TL = tibia length, BPM = beats per minute.

**S8**

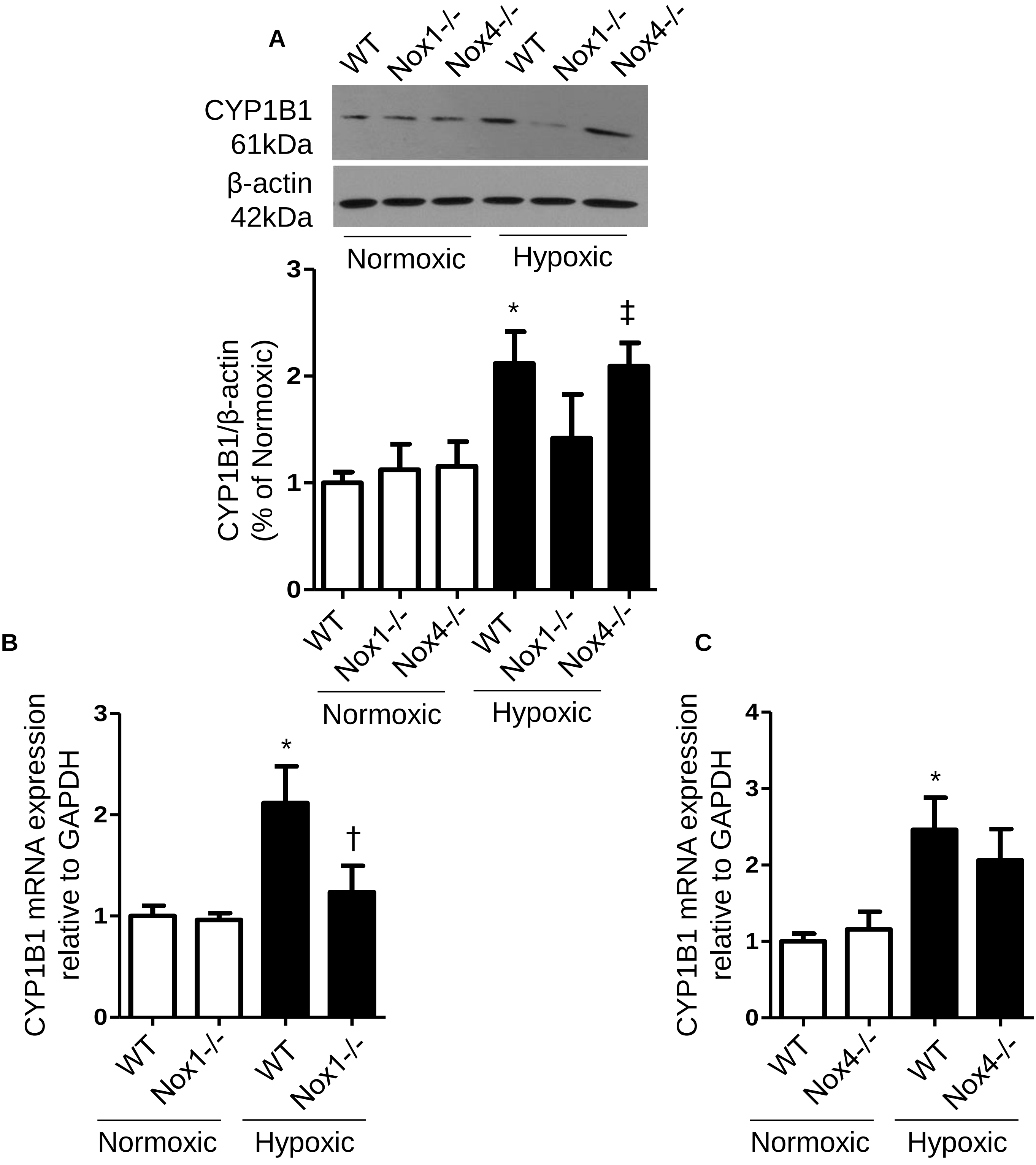


**S8: CYP1B1 mRNA and protein expression in Nox1-/- and Nox4-/- mouse pulmonary artery tissue.** Protein expression of CYP1B1 in pulmonary arteries of WT, Nox1-/- and Nox4-/- mice (A). Transcript levels of CYP1B1 in female WT and Nox1-/- mouse pulmonary artery tissue (B) and in WT and Nox4-/- mouse pulmonary artery tissue (C). Results are mean ±SEM of 4 experiments, in triplicate. Graphs represent the protein expression relative to β-actin or mRNA expression relative to GAPDH. *$p<0.05$, vs. normoxic WT; ‡$p<0.05$, vs normoxic Nox4-/-; †$p<0.05$, vs. hypoxic WT, determined by ANOVA with Tukey's post-hoc test.
